# Characteristics of cyclist crashes in Italy using latent class analysis and association rule mining

**DOI:** 10.1371/journal.pone.0171484

**Published:** 2017-02-03

**Authors:** Gabriele Prati, Marco De Angelis, Víctor Marín Puchades, Federico Fraboni, Luca Pietrantoni

**Affiliations:** Department of Psychology, University of Bologna, Bologna, Italy; Beihang University, CHINA

## Abstract

The factors associated with severity of the bicycle crashes may differ across different bicycle crash patterns. Therefore, it is important to identify distinct bicycle crash patterns with homogeneous attributes. The current study aimed at identifying subgroups of bicycle crashes in Italy and analyzing separately the different bicycle crash types. The present study focused on bicycle crashes that occurred in Italy during the period between 2011 and 2013. We analyzed categorical indicators corresponding to the characteristics of infrastructure (road type, road signage, and location type), road user (i.e., opponent vehicle and cyclist’s maneuver, type of collision, age and gender of the cyclist), vehicle (type of opponent vehicle), and the environmental and time period variables (time of the day, day of the week, season, pavement condition, and weather). To identify homogenous subgroups of bicycle crashes, we used latent class analysis. Using latent class analysis, the bicycle crash data set was segmented into 19 classes, which represents 19 different bicycle crash types. Logistic regression analysis was used to identify the association between class membership and severity of the bicycle crashes. Finally, association rules were conducted for each of the latent classes to uncover the factors associated with an increased likelihood of severity. Association rules highlighted different crash characteristics associated with an increased likelihood of severity for each of the 19 bicycle crash types.

## Introduction

In Europe, bicycle is the most frequently used mode of transport for 8% of people. In Italy, 6% of people report that bicycle is the most frequently used mode of transport [1]. Cycling is considered a healthy [2–6] as well as an environmentally friendly mode of transportation [7–9]. For these reasons, the promotion of bicycle use has received an increasing interest among policy-makers. However, safety concerns associated with bicycle use constitute a possible barrier to the wide-scale promotion of bicycle use [10, 11].

There is a growing interest in the literature in understanding the risk factors of bicycle crashes to design and implement the most effective countermeasures to reduce the risk and the severity of bicycle crashes. According to a conceptual framework for road safety and mobility applied to cycling safety [11], bicycle crash is a function of three ‘safety pillars’: infrastructure, road user(s) and vehicle(s).

Infrastructure refers to road characteristics such as type of road section or location (rural vs. urban). There is evidence that bicycle crashes are more likely in urban or arterial roads [12–16]. However, the severity of bicycle crashes is higher in rural roads than in urban roads [17–20]. A greater risk of bicycle crashes has been found at intersection compared to other road sections [12, 16, 21–24]. Furthermore, roundabouts have been found to increase the risk of bicycle crashes [12, 25]. In terms of bicycle crash severity, bicycle crashes in straight sections are the most severe [26, 27], while bicycle crashes occurring at signalized intersections are less severe than those occurring elsewhere [28]. Finally, although most bicycle crashes tend to occur on dry surfaces [29, 30], the presence of hazards on the road surface, such as sand, gravel, steepness, road curves, uneven or wet surface, is likely to increase the risk of collision [31–33].

The category of road users refers to both road users’ characteristics (e.g., age and gender) and their behavior (e.g., maneuvers, speed choice, violations). Among the most investigated types of maneuvers or violations associated with bicycle crashes, we can mention not respecting the traffic signals or failing to properly yield at an intersection by both the cyclist [16, 32–39] and the driver of the opponent vehicle [16, 25, 32–38, 40–43]. In addition, gender and age were found to influence the likelihood of being involved in crashes [33, 44]. Moreover, older or male cyclists were more likely to sustain a fatal injury than younger or female cyclists, respectively [18, 19, 23, 26, 28, 33, 41, 45–50].

The category of vehicle refers the characteristics of the vehicle itself. A higher risk of collision has been found to be associated with increasing levels of van, large automobile, and truck traffic [25, 51]. Furthermore, the likelihood of fatal crash increases when a large vehicle (i.e., truck or bus) is the opponent vehicle in a bicycle crash [33, 47, 52]. Compared to cars, large vehicles (i.e., truck or bus) have more blind spots and when a cyclist is in the blind spot of a large vehicle a higher risk of collision is expected [29, 52].

In addition to these three safety pillars, the season and weather conditions may have an influence on bicycle crashes [14, 29]. Also, there is evidence that most bicycle crashes occur in urban areas, on weekdays, and during daylight [29, 30, 53]. However, bicycle crashes occurring at day-time under good visibility tend to be less severe [26–28, 33, 47]. Also, the severity of bicycle crashes has been associated with inclement weather [47] and foggy weather [27].

It is unclear, however, whether the magnitude of the effect of risk factors is different for different conditions. For instance, the influence of a specific risk factor may be insignificant when analyzing the whole bicycle crash data but, at the same time, it may be highly influential for a specific type of bicycle crash. Indeed, bicycle crashes occur under different conditions and bicycle crash data are highly heterogeneous in nature. Thus, it is necessary to distinguish the typology of bicycle crashes to avoid making data heterogeneous. By taking into account the systematic heterogeneity in bicycle crashes, researchers and practitioners can identify the most appropriate safety countermeasures for different bicycle crash types occurred under different conditions. To reduce heterogeneity, researchers focused on narrow bicycle crash characteristics such as type of opponent vehicle [54] or type of location [55]. Although the findings of these studies are important to identify the risk factors and the appropriate safety countermeasures by reducing heterogeneity, this approach does not guarantee that bicycle crashes data comprise homogeneous groups of bicycle crashes.

One way to account for the heterogeneous nature of the data is to use a latent class clustering approach. Latent class analysis can segment the bicycle crash data into mutually exclusive and exhaustive latent classes by assuming a latent categorical variable. The class memberships of each bicycle crashes can be inferred from the observed variables.

The current study contributes to the literature on bicycle crashes by identifying the homogenous bicycle crash groups in Italy through a latent class clustering approach. A previous study on cyclist–motorist crashes in Denmark suggests that a latent class clustering approach can be useful for studying bicycle crashes [29]. Specifically, the researchers found 13 distinguishable types of cyclist–motorist crashes. Several variables contributed to the identification of the latent classes: motorized vehicle pre-crash maneuvers, availability of a cycle lane, speed limit, number of lanes, infrastructure type, road surface conditions, cyclist intoxication, and helmet wearing behavior. Based on the analysis of these 13 bicycle crash patterns, three types of safety considerations were proposed. These safety considerations related to network design and connectivity, road maintenance, and cyclist road behavior.

### The present study

The current analysis focused on cyclist crashes that occurred in Italy during the period between 2011 and 2013. To identify reliable and relevant subgroups of bicycle crashes, we used latent class analysis. Latent class analysis allows identification of categorical latent variables (called “latent classes”) in categorical cross-sectional observations. Latent class analysis can be applied on polytomous manifest variables such as the categorical indicators corresponding to the characteristics of infrastructure, road user(s), vehicle(s), and the environmental and light conditions. Specifically, characteristics of infrastructure (road type, road signage, and location type), road user (i.e., opponent vehicle and cyclist’s maneuver, type of collision, age and gender of the cyclist), vehicle (type of opponent vehicle), environmental and time period variables (time of the day, day of the week, season, pavement condition, and weather) were employed in the analysis. Data concerning cyclist injury severity were not included in latent class analysis because cyclist injury severity is considered an outcome of the crash [29]. Rather, we investigated the relationship between different latent classes and cyclist injury severity using logistic regression analysis. This analysis allows for the identification of the most dangerous subgroups of bicycle crashes.

To identify the main factors that contribute to bicycle crash severity within each subgroup of bicycle crashes, we used association rule algorithms for each one of the latent classes. To identify factors associated with the severity of bicycle crashes, different types of analysis such as the generalized linear model of logistic regression, binary logit model, multinomial logit model, and mixed logit model have been employed [56]. However, the use of regression models has clear shortcomings because of the mass of complicated data on road accidents. Specifically, regression models involve strong statistical assumptions rarely met in real accidents data. Moreover, regression models may not satisfactorily (1) detect interaction that may occur in complex forms and (2) handle many discrete variables or variables with a high number of categories. Association rules provide insight and permits of easily investigate associations between any of the bicycle crash attributes. Furthermore, association rule algorithms can find patterns within a potentially large number of categorical variables or variables with a high number of categories and, therefore, do not rely on different and strong statistical assumptions (e.g., no outliers, linearity in modeling the relationship).

This manuscript presents an analysis of bicycle crashes based on a combination of latent class analysis, logistic regression, and association rule algorithms. To our knowledge, this is the first study in which this combination of latent class analysis and data mining approach has been employed in literature on bicycle crashes.

## Method

### Data

The crash data we used to estimate the models were provided by the Italian National Institute of Statistics (ISTAT). The ISTAT collects and provides with all road crashes gathered by the following different collaborating public institutions: The Italian Ministry of Transport and Infrastructure, local Municipalities, National Police agencies, the Italian Automobile Club, as well as the ISTAT.

**Fig 1:**
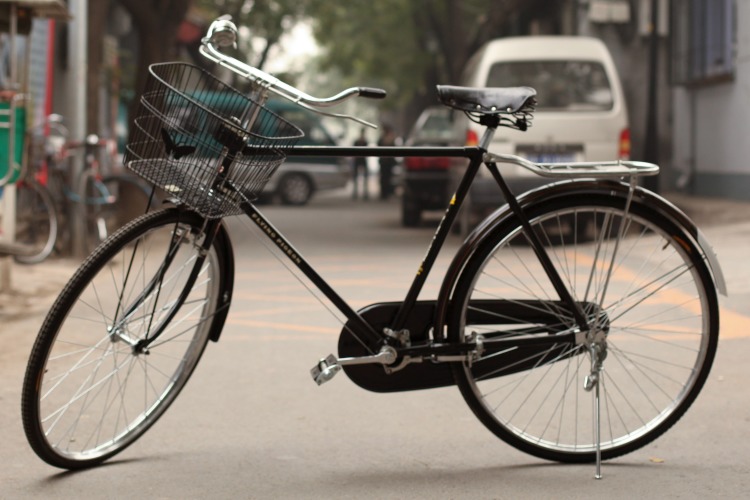
Human powered bike.

**Fig 2:**
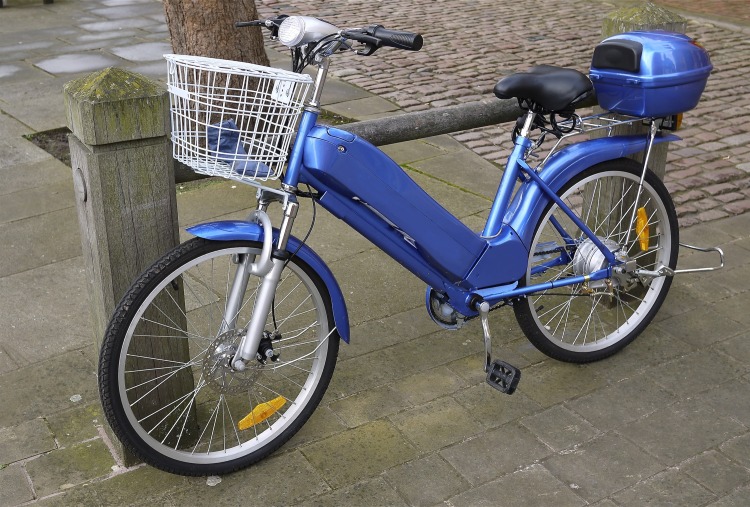
Electrically powered bike.

The original database comprised 575,093 road accidents that took place from 2011 to 2013 in Italian roads. At the time of the study, 2013 was the most recent available ISTAT data. Depending on Italian Highway Code, e-bikes (e.g., pedelecs) are legally classified as bicycles. Therefore, in the present study, bicycles are considered as both human-powered bicycle and electrically powered bicycle. Figs 1 and 2 display an example of each of these types of bicycles. We chose a three-year period because we decided to have a trade-off between the need to have a large sample size and the need to control for change in road regulation. Indeed, in 2010 (Law L. 29/7/2010 n. 120) a new national traffic law was approved, with minor changes involving also bicycle use. To narrow down the events to those pertinent for the current research, we extracted the 49,621 accidents in which at least one cyclist ended up injured or killed. The ISTAT database makes difference between those road accidents resulting in injuries or fatalities (within 30 days), nevertheless, it does not distinguish among different levels of injury. As Table 1 shows, we selected 15 categorical variables in the resulting database: (1) type of opponent vehicle, (2) opponent vehicle manoeuver, (3) road type, (4) pavement condition, (5) cyclist’s age, (6) cyclist’s gender, (7) cyclist’s maneuver, (8) type of collision, (9) time of the day, (10) day of the week, (11) season, (12) weather, (13) road signage, (14) accident location type, and (15) severity of bicycle crash. According to the Italian Road Code, national, regional, and provincial roads crossing urban communities with less than 10,000 inhabitants are considered as urban national, urban regional and urban provincial roads, respectively. Furthermore, concerning the weather condition category, the checklist used by Italian authorities to collect accident data considers as clear weather all those weather conditions that does not involve inclement weather (e.g. rainy, snow, etc.). Therefore, a bicycle crash happened in a sunny or cloudy condition has been classified into the clear weather category. Finally, based on a previous study [28], we partitioned into three time periods: day time (6 a.m.–6 p.m.), evening (6 p.m.–midnight), and late night (midnight–6 a.m.).

**Table 1:** Sample Characteristics.

Variable	*N*	%
Opponent vehicle		
Car	35246	71.0
Bus	365	0.7
Truck	3050	6.1
PTW	2952	5.9
Other vehicles	945	1.9
Multiple vehicles	910	1.8
No opponent vehicle	6153	12.4
Opponent vehicle maneuver		
Straight forward or normal driving	11030	22.2
Not keeping a safe distance	2795	5.6
Ignoring stop signs or red traffic light	2857	5.8
Not respecting the right of way	7028	14.2
Driving in a forbidden direction or on opposite side of road	540	1.1
Traveling too fast	1626	3.3
Turning right	1661	3.3
Turning left	2357	4.8
Overtaking	747	1.5
Unknown or others	18980	38.2
Road type		
Urban municipal	39327	79.3
Urban provincial, regional and national	4540	9.15
Rural	5754	11.6
Pavement condition		
Dry	45079	90.8
Wet	4178	8.4
Slippery, frozen, or snowy	364	0.7
Cyclist’s age		
0–14	3142	6.3
15–24	5919	11.9
25–44	14550	29.3
45–54	7974	16.1
55–64	6236	12.6
65 and older	11504	23.2
Not specified	296	0.6
Cyclist’s gender		
Male	33912	68.3
Female	15709	31.7
Cyclist’s maneuver		
Straight forward or normal driving	21247	42.8
Not keeping a safe distance	1439	2.9
Ignoring stop signs or red traffic light	1628	3.3
Not respecting the right of way	2112	4.3
Driving in a forbidden direction or on opposite sides of road	3599	7.3
Traveling too fast	858	1.7
Turning right	364	0.7
Turning left	1626	3.3
Overtaking	317	0.6
Unknown or others	16431	33.1
Type of collision		
Head-on collision	3202	6.5
Side-impact	34693	69.9
Rear-end collision	3920	7.9
Hit pedestrian	257	0.5
Hit stopped vehicle	2721	5.5
Hit parked vehicle or object	1122	2.3
Run-off-the-road	1912	3.9
Other (no vehicle was involved)	1735	3.6
Time of the day		
Daytime (6.00 am to 6.00 pm)	40676	82.0
Evening (6.00 pm to midnight)	7881	15.9
Late night (midnight to 6.00 am)	898	1.8
Not specified	166	0.3
Day of the week		
Weekdays	39027	78.7
Weekend	10549	21.3
Season		
Winter	8034	16.2
Spring	14783	29.8
Summer	15736	31.7
Autumn	11068	22.3
Weather		
Clear	44072	88.8
Foggy	267	0.5
Rainy	2381	4.8
Hail, Snow, Strong wind, other	2901	5.8
Road signage		
Absent	4171	8.4
Vertical	3265	6.6
Horizontal	3988	8.0
Vertical and horizontal	38197	77.0
Location type		
Crossroads	22294	44.9
Not at junction	22903	46.2
Roundabouts	4424	8.9
Severity of bicycle crash		
Injury	48798	98.3
Fatality	823	1.7

### Statistical analysis

To perform latent class analysis, we used the software *R* [57] with the package poLCA [58]. The package poLCA allows for the estimation of latent class model for polytomous outcome variables. The latent class model analyses *J* polytomous categorical variables, each of which comprises *K*_j_ possible outcomes, for observations *i* = 1…*N*. The indexing by *j* is due to the wide range of outcomes of the categorical variables. The observed values of the *J* categorical variables are represented as *Y*_ijk_ such that *Y*_ijk_ = 1 if the observation *i* provides the *k*th response to the *j*th variable, otherwise Y_ijk_ = 0, where *j* = 1…*J* and *k* = 1…*K*_j_. The latent class model estimates the observed joint distribution of the categorical variables in the observations, and approximates it as the weighted sum of a finite number, *R*, which is formed by a large multi-way contingency table. To determine an appropriate number of latent classes *R* for the present data set, we, first, fitted a complete “independence” model with *R* = 1, and then iteratively increase the number of latent classes by one until an appropriate fit has been reached. Denote as π_jrk_ the class-conditional probability that an observation in class *r* = 1…*R* determines the *k*th outcome on the *j*th variable. Consequently, within each class, for each manifest variable

$$\sum_{k=1}^{K_{j}}\pi_{jrk}=1$$

Moreover, let *p*_r_ denote the R mixing proportions that produce the weights in the weighted sum of the component tables, with ∑_*r*_ *p*_*r*_ = 1. The result of the following equation produces the likelihood that an observation *i* in class *r* determines a particular set of *J* outcomes on the polytomous categorical variables, assuming local independence:

$$f(Y_{i};\;\tau_{r})=\prod_{j=1}^{J}\prod_{k=1}^{Kj}{\left(\pi_{jrk}\right)}^{Y_{ijk}}$$

The weighted sum produces the probability density function across all classes:

$$Pr(Y_{i}|\pi ,p)=\sum_{r=1}^{R}p_{r}\prod_{j=1}^{J}\prod_{k=1}^{K_{j}}{\left(\pi_{jrk}\right)}^{Y_{ijk}}$$

The latent class model estimates the parameters *p*_r_ and π_jrk_. With respect to estimates ${\hat{p}}_{r}$ and ${\hat{\pi }}_{jrk}$ of *p*_r_ and π_jrk_, the following Bayes' formula has been used to calculate the posterior probability that each observation belongs to each class, conditional on the observed values of the categorical variables:

$$\hat{Pr}(r|Y_{i})=\frac{{\hat{p}}_{r}f(Y_{i};{\hat{\pi }}_{r})}{\sum_{q=1}^{R}{\hat{p}}_{q}f(Y_{i}f(Y_{i};{\hat{\pi }}_{q})}$$

It is important to remain aware that the ${\hat{\pi }}_{jrk}$ are estimates of outcome probabilities conditional on class *r*. The latent class model is estimated by poLCA by maximizing the log-likelihood function

$$lnL=\sum_{i=1}^{N}ln\sum_{r=1}^{R}p_{r}\prod_{j=1}^{J}\prod_{k=1}^{K_{j}}{\left(\pi_{jrk}\right)}^{Y_{ijk}}$$

with respect to the parameters *p*_r_ and π_jrk_. The expectation-maximization algorithm was applied to the log-likelihood function by treating as missing data each unknown observation class membership.

We fitted separately and compared twenty class models using Akaike Information Criterion (AIC), consistent AIC (CAIC), the Bayesian Information Criterion (BIC), and the adjusted BIC (ABIC). The BIC has been found to be a good indicator for the determination of the optimal number of classes, and a better statistical indicator than the AIC [59]. The rule of the smaller the better should be applied to evaluate the fit indices. To ensure that the global maximum likelihood of the latent class model has been achieved (rather than local maximum log-likelihood), we automatically re-estimated each model ten times and saved the model with the greatest likelihood.

Given our aim of identifying the most dangerous classes of accident patterns, we dummy coded the different classes (i.e., for each latent class, the value 0 equals not belonging to that class and 1 equals belonging thereto) to be able to estimate the increase in odds ratio of fatality associated with belonging to each class. We employed logistic regression using the backwards stepwise method of field selection to exclude from the model the classes not accounting for variations in odds of the fatal outcome. To investigate the factors related to the severity of bicycle crash, an association rules algorithm was used. Compared to other data mining techniques, such as decision tree algorithms, association rules algorithms have the advantage of finding associations between any of the attributes of the variables. While a decision tree algorithm is able to build rules with only a single conclusion, association rules algorithms can find many rules, each of which may result in a different conclusion. Therefore, it can discover a wide range of interactions by investigating all combinations of variables. To discover association rules in the data, we used the Apriori algorithm. Regarding the methods of selecting rules, we chose confidence ratio because this method takes uneven distributions into account. Therefore, it is appropriate to find rules that predict rare events such as bicycle fatality crashes. To identify rules of interest, we selected the five two-item rules with the highest confidence. In line with a previous study on road accidents [60], we chose a minimum support value of 5%. Both logistic regression and associations rules were carried out using IBM SPSS Modeler v.18.

## Results

As reported in Table 1, the opponent vehicle was car in 71.0% of bicycle accidents, followed by truck (6.1%), powered two-wheelers (PTW; 5.9%), and bus (0.7%). To understand how these statistics differ from non-accident involved statistics, we retrieved road vehicle statistics from Eurostat (http://ec.europa.eu/eurostat/web/transport/data/database). According to the data provided by Eurostat, in Italy, in the period 2011–2013, passenger cars make up about 78% of stock of vehicles, followed by PTW (about 13%), truck (9%), and bus (about 0.2%).

To determine the most appropriate number of latent classes, we made a comparison between the four different criteria (AIC, BIC, ABIC and CAIC). Analysis of data indicated that the 19-class model was the best fitting solution as compared to the other models (Table 2). Specifically, the 19-class solution had lowest BIC, ABIC and CAIC. Although the model with the lowest AIC was the 20-class solution, the percentage decrease in AIC drops from the 19-class solution to the 20-class solution is negligible (0.04%). Moreover, the BIC, ABIC and CAIC have a clear superiority to the AIC [59]. Therefore, the 19-class model was chosen. To assess accuracy in the classification, we used the average posterior probabilities to compute the odds of correct classification for each class. S1 Table shows the confusion matrix that demonstrates a good classification accuracy. Specifically, all of the clusters were correctly identified for 83% of the observations.

**Table 2:** Values of AIC, BIC, aBIC, and CAIC as a Function of the Number of Latent Classes.

Model	AIC	BIC	aBIC	cAIC
1-Class	1328680	1329182	1329001	1329239
2-Class	1275106	1276119	1275754	1276234
3-Class	1242662	1244186	1243636	1244359
4-Class	1225864	1227900	1227166	1228131
5-Class	1214077	1216624	1215706	1216913
6-Class	1205537	1208594	1207492	1208941
7-Class	1201838	1205407	1204120	1205812
8-Class	1197923	1202003	1200531	1202466
9-Class	1195906	1200497	1198841	1201018
10-Class	1192648	1197750	1195910	1198329
11-Class	1191447	1197060	1195036	1197697
12-Class	1189229	1195353	1193144	1196048
13-Class	1188256	1194892	1192499	1195645
14-Class	1186825	1193972	1191394	1194783
15-Class	1186455	1194113	1191351	1194982
16-Class	1184919	1193088	1190142	1194015
17-Class	1184225	1192905	1189774	1193890
18-Class	1183211	1192402	1189087	1193445
19-Class	1182634	1192336	1188837	1193437
20-Class	1182598	1192811	1189128	1193970

**Fig 3:**
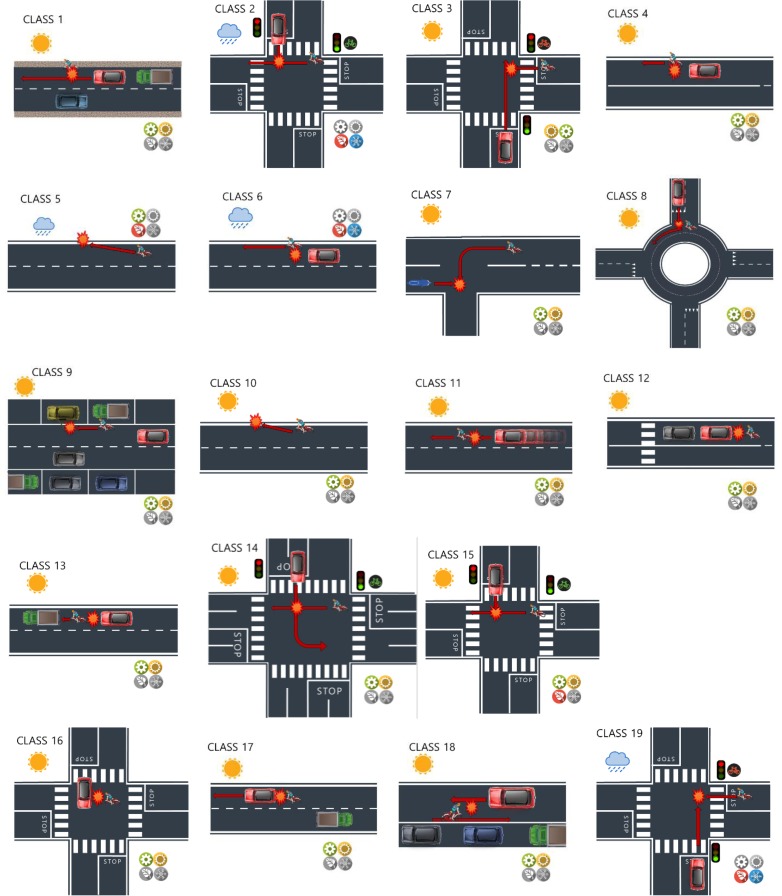
Representation of the 19 types of bicycle crashes.

S1 Table shows the proportion of individuals classified into each latent class as well as the conditional response probabilities. Conditional response probabilities refer to the probability that a bicycle crash of a certain latent class exhibits a certain value of a variable. For example, a conditional response probability of .60 for daytime and the first latent class would reflect that 60% of the bicycle crashes within the first latent class did happen during daytime. We now proceed to characterize each one of the latent classes displayed in S2 Table and further represented graphically in Fig 3.

Class 1 (C1) has a 12.2% of accidents against a truck and an 11.0% against multiple vehicles. The highest group of age represented was that from 45 to 54 years-old (22.1%). The most common maneuver was unknown or not specified (77.6%). The opponent vehicle’s most typical maneuver was driving straightforward (48.6%). The majority of the accidents happened during spring (31.1%) or summer (36.6%). The majority of the cases involved dry pavement (96.1%), clear weather (94.6%), an impact on the side (55.1%), and did not happen at junction (82.5%).

Class 2 (C2) is one of the few clusters featuring more than 20% of accidents in the evening (20.6%). The most common cyclist’s maneuver was riding straightforward (84.9%), whereas that of the driver consisted in not respecting the right of way (59.9%). The majority of the accidents happened during autumn (41.6%) or winter (32.0%). Moreover, the pavement was typically wet (97.2%), it was raining (64.0%), the impact was on the side (93.8%), and the accident took place at a crossroad (73.3%).

Class 3 (C3) is characterized by a combination of accidents involving cyclists’ maneuvers such as ignoring a red traffic light or stop signs (22.3%), not respecting the right of way (28.5%), and cycling in a forbidden direction or in opposite sides of the road (21.1%). The opponent vehicle was typically driving straightforward (74.0%). The most of its accidents happened in spring (31.7%) or summer (34.2%). The pavement was typically dry (99.8%), the weather was clear (94.8%), the collision was on the side (90.8%), and the accident took place at intersection (91.3%).

Class 4 (C4) features a majority of accidents where the cyclist rode straightforward (91.5%) and the opponent vehicle’s maneuver was unknown (74.5%). The majority of the accidents took place in spring (31.5%) or summer (32.3%). The pavement was typically dry (97.9%), the weather was clear (95.1%), the impact was from the side (90.4%), and the collision did not take place at junction (94.0%).

Class 5 (C5) is characterized by being one of the few classes with more than 20% of the cyclists ranging from 45 to 54 years-old (20.4%). The cyclist’s maneuver was typically unknown (85.2%), and there was no opponent vehicle (100%). The majority of the accidents took place during spring (25.0%) or autumn (37.0%). The ground was wet most of the times (97.2%), it was raining (53.7%), the collision was typically the result of a cyclist running off the road (44.7%) or happened for not specified reasons (33.9%), and it did not occur at junction (70.5%).

Class 6 (C6) is characterized by a 40.2% of the cyclists involved ranging from 25 to 44 years-old. A 41.7% of the cyclists were riding straightforward and the maneuvers of a 46.9% of the cyclists were unknown. Regarding the opponent vehicle, the majority (56.2%) of the maneuvers were also unknown. The majority of the accidents took place in autumn (42.9%) and winter (30.2%). The pavement was typically wet (98.2%), it was raining (70.2%), the collision was from the side (68.5%), and did not happen at junction (73.4%).

Class 7 (C7) features 31.5% of accidents occurring against a PTW, and a 33.0% of the accidents involving cyclists aged 65 years or older. The cyclist’s maneuver is typically turning left (77.0%), and that of the opponent vehicle was going straight forward (68.1%). The majority of the accidents took place in spring (31.6%) or summer (36.7%), the pavement was dry (97.7%), the weather was clear (95.0%), and the impact was from the side (89.8%).

In the accidents comprised in Class 8 (C8), the cyclist was typically riding straightforward (88.9%), and the opponent vehicle did not respect the right of way (71.0%). The crashes mostly took place during spring (27.9%) or summer (30.7%), when the pavement was dry (99.5%), and the weather was clear (93.2%). The impact was typically on the side (95.5%), and it took place at roundabouts (70.4%).

Class 9 (C9) features bicycle accidents without an opponent vehicle (100%), and cyclists mostly riding straightforward (49.8%) or performing an unclassified maneuver (26.2%). The majority of the accidents took place during spring (34.6%) or summer (37.1%), when the pavement was dry (97.3%), and the weather was clear (95.9%). The collision typically involved hitting an object (44.7%), and the accident did not happen at junction (71.9%).

In Class 10 (C10), the 100% of the accidents were single crashes (i.e., there was no opponent vehicle), and the majority of the cyclists’ maneuvers were unknown (97.1%). The majority of the accidents took place during spring (32.5%) or summer (35.3%), the pavement was dry (96.4%), clear weather (95.2%), the collision involved running off the road (46.8%) or another event not categorized (44.9%), and did not take place at a junction or at a roundabout (78.0%).

Class 11 (C11) features 92.1% of the cyclists riding straightforward. The opponent vehicles were typically not respecting the safety distance (51.7%) or speeding (30.5%), and most of the accidents happened during spring (28.3%) or summer (36.6%). Moreover, in the majority of the bicycle crashes, the pavement was dry (98.0%), the weather was clear (94.8%), the type of collision was on the rear end (64.5%), and the location was not at a junction or at a roundabout (69.7%).

Class 12 (C12) is one of the few classes with more than 40% female cyclists (41.9%). The cyclist’s (73.3%) and the opponent vehicle’s (80.0%) maneuvers were mostly unknown. The majority of the accidents took place during spring (31.2%) or summer (30.4%), when the ground was dry (98.11%), and the weather was clear (95.5%). The majority of the cyclists hit a stopped vehicle (94.5%), and did not take place at a junction or at a roundabout (88.8%).

Class 13 (C13) is also one of the few clusters with more than 40% of female cyclists (42.1%). The majority of the cyclists were riding straightforward (81.2%), and the opponent vehicle was not keeping the safety distance (95.5%). The most of the accidents happened during spring (28.8%) or summer (30.2%), when the pavement was wet (96.0%), and the weather was clear (96.3%). The most of the collisions were with a stopped vehicle (69.5%), and they did not occur at a junction or at a roundabout (89.4%).

In Class 14 (C14), 33.4% of the cyclists’ age ranged from 25 to 44 years-old. The majority of the cyclists’ maneuvers involved riding straightforward (82.8%), and the opponents’ maneuvers involved not respecting the right of way (46.0%) and ignoring a stop sign or red traffic light (27.5%). Most of the accidents took place during spring (29.6%) or summer (27.0%), with clear weather (94.4%), the pavement was dry (99.5%), the impact was on the side (90.5%), and it did take place at a crossroad (95.8%).

Class 15 (C15) is one of the few clusters with more than 40% of women (46.0%) among the cyclists. Most common cyclist’s maneuvers consisted in riding straightforward (81.8%), and the most common maneuvers by the opponent vehicle were not respecting a red traffic light or a stop sign (26.2%) and not respecting the right of way (42.4%). Moreover, between 25 and 30% of the accidents took place during spring, summer or autumn. The ground was typically dry (99.0%), the weather was clear (93.5%), the impact was on the side (90.9%), and the accidents took place at crossroads (99.9%).

Class 16 (C16) features a majority of cyclists (99.2%) and opponent vehicles (85.1%) taking unknown maneuvers. The majority of the accidents took place during spring (28.6%) or summer (35.2%), the pavement was dry (98.1%), it was sunny (92.9%), and the collision was on the side (84.7%). Finally, 48.3% of the accidents took place at crossroad, and 39.8% did not take place at junction.

In Class 17 (C17), 35.6% of the cyclists were aged between 25 and 44 years. The most common cyclist’s maneuver involved not keeping the safety distance (92.4%), whereas the most common maneuver of the opponent vehicle was that of driving straightforward (95.5%). In addition, the most of the accidents took place during spring (34.0%) or summer (28.1%), the road was dry (96.8%), and the weather was clear (94.9%). Finally, the majority of the collisions were on the rear end (35.9%) or hitting a stopped vehicle (45.2%), and the 77.5% of the accidents did not take place at junctions.

Class 18 (C18) features accidents where the majority of the cyclists’ maneuvers were unknown (73.4%), and the opponent vehicles’ maneuvers were mostly driving straightforward (74.9%). The majority of the accidents took place in spring (34.0%) or summer (33.9%), the pavement was generally dry (97.9%), the weather was clear (95.1%), the collision was on the side (87.0%), and the accidents did not take place at a junction or at a roundabout (99.8%).

Finally, Class 19 (C19) is one of the few clusters that comprised more than 20% of the accidents that happened in the evening (21.7%). In this class, the most common cyclist’s maneuvers were ignoring stop signs or traffic lights (20.6%), not respecting the right of way (21.8%), and driving in a forbidden direction (23.4%), whereas the opponent vehicle’s most common maneuver was driving straightforward (74.5%). Moreover, the most of the accidents took place during autumn (40.6%) or winter (28.8%), when the pavement was wet (97.9%), and typically raining (58.7%). Most of the collisions were on the side (89.4%), and the majority of the accidents occurred at crossroads (78.2%).

Table 3 displays the findings of logistic regression analysis predicting the severity of bicycle crashes. C1 and C11 are the most important predictors of the severity of bicycle crashes. Indeed, C1 and C11 have the highest cyclists’ fatality share, with percentages of 6.5 and 5.6, respectively, in comparison with the average fatality share of 1.7 percent in the whole data set. On the contrary, eight latent classes (C2, C5, C8, C9, C12, C13, C15, and C19) were not included in the final model (Table 3) using the backwards stepwise method. The bicycle crashes included in these eight latent classes have a fatality share lower than 1%.

**Table 3:** Summary of Logistic Regression Analysis Predicting the Severity of Bicycle Crashes.

	*B*	*SE*	Wald	*p*	*OR*	95% CI
C1	2.27	0.15	243.39	< .001	9.701	[7.292, 12.906]
C3	0.76	0.14	31.93	< .001	2.147	[1.647, 2.798]
C4	0.58	0.15	14.56	< .001	1.793	[1.328, 2.421]
C6	0.88	0.23	14.71	< .001	2.409	[1.537, 3.775]
C7	1.05	0.26	16.14	< .001	2.859	[1.713, 4.773]
C10	1.24	0.14	75.44	< .001	3.471	[2.621, 4.596]
C11	2.12	0.12	295.44	< .001	8.301	[6.521, 10.567]
C14	0.50	0.21	5.60	.018	1.647	[1.089, 2.489]
C16	0.78	0.16	23.42	< .001	2.187	[1.593, 3.002]
C17	0.80	0.31	6.81	.009	2.216	[1.219, 4.029]
C18	0.92	0.15	38.08	< .001	2.502	[1.87, 3.348]

CI = confidence interval for odds ratio (*OR*).

S3 Table shows the findings of association rules using the Apriori algorithm with severity (bicycle crashes resulting in fatalities) as consequent for each of the latent classes. Association rules are statements in the form: if antecedent(s) then consequent(s). Thus, association rules associate the fatal bicycle crashes with a set of conditions or antecedents. Support, confidence, and lift provide information about the rules. Support refers to the size (percentage) of the subsets of the bicycle crashes for which the antecedents (the ‘if’ part of the rule) are true. Confidence refers to the proportion of bicycle crashes with the specified antecedent(s) for which the consequent (i.e., fatality) is also true. Therefore, confidence is the percentage of bicycle crashes resulting in fatalities within the bicycle crashes that contain all the antecedents. Lift is based on the ratio of confidence for the rule to the prior probability of having a fatality crash within that subgroup of bicycle crashes.

Association rules for C1 showed that fatal bicycle crashes were more likely at evening hours and when (1) the type of collision was rear-end, or (2) the opponent vehicle was a car, or (3) the season was spring, or (4) vertical and horizontal road signage was present. Also, cyclists aged 65 years or older cycling on weekdays were more likely to have a fatal crash. Association rules for C2 indicated that fatal bicycle crashes were more likely when the cyclist’s age was 65 years or older and (1) the season was autumn, or (2) the gender of cyclists was male, or (3) the cyclist’s maneuver was straight forward or normal driving, or (4) vertical and horizontal road signage was present, or (5) not respecting the right of way was the maneuver of the opponent vehicle. Association rules for C3 showed that fatal bicycle crashes were more likely in a rural road and when (1) the opponent vehicle was a car, or (2) the cyclist gender was male, or (3) the crash took place at a crossroad, or (4) vertical and horizontal road signage was present, or (5) the weather was clear. Association rules for C4 indicated that fatal bicycle crashes were more probable when (1) the type of collision was head-on and the gender of cyclists was male; (2) the road was rural and vertical and horizontal road signage was present or the maneuver of the opponent vehicle was unknown or others; (3) the season was autumn and the age of the cyclist was 65 or older; and (4) the opponent vehicle was a truck and the gender of cyclists was male. Association rules for C5 showed that rural road incremented the likelihood of fatal bicycle crash. Also, rural road was associated with a higher probability of fatal bicycle crash when (1) the gender of cyclists was male, or (2) the crash did not take place at a junction or at a roundabout, or (3) the cyclist maneuver was unknown or other. Rules revealed that when the cyclist’s age was between 45 and 54 years and the weather was clear, fatal bicycle crashes were more likely. Association rules for C6 indicated that fatal bicycle crashes were more probable when the maneuver of the opponent vehicle was traveling too fast during daytime. Rules also reveal that rural roads were associated with fatal bicycle crashes especially when (1) the cyclist’s maneuver was unknown or other, or (2) the cyclists’ age was between 25 and 44 years, or (3) the crashes happened on weekdays, or (4) the opponent vehicle was a car. Association rules for C7 showed that fatal bicycle crashes were more likely during the weekend when (1) the cyclist’s age was 65 years or more, or (2) the opponent vehicle was a car, or (3) during daytime, or (4) at crossroads. Rules also revealed that rural roads were associated with fatal bicycle accidents when the cyclists were female. Association rules for C8 indicated that fatal bicycle accidents were more probable when the opponent vehicle was a truck and (1) the cyclists were riding straight forward, or (2) the bicycle crash occurred on weekdays, or (3) the bicycle crash occurred at daytime, or (4) the bicycle crash occurred with a clear weather, or (5) the type of collision was a side-impact. Association rules for C9 show that fatal bicycle accidents mainly occurred on urban roads when (1) the road signage was absent and the gender of the cyclist was male or (2) hit a parked vehicle. Association rules also revealed that cyclist’s deaths were more likely when the cyclist’s age was 65 or more years and the cyclist’s gender was male; in addition, bicycle crashes that occurred on rural roads, on location types that are not junctions, were more prone to bicyclist fatalities. Association rules for C10 showed that fatal bicycle accidents were more likely to occur on rural roads during weekdays. Association rules also showed that fatal bicycle crashes were more probable when the cyclist’s gender was male or the cyclist’s age was 65 or more years or the bicycle crash occurred on rural roads. Association rules for C11 indicated that fatal bicycle crashes were more likely when the bicycle crash took place on rural roads. In addition, the severity of bicycle crashes increased when the opponent vehicle was a car which was traveling too fast. Association rules also showed that on rural roads, the cyclist’s death was more likely when the cyclist (1) was 65 or more years old and (2) was riding through a crossroads, and (3) was cycling during the evening. Association rules for C12 showed that fatal bicycle accidents were more probable on urban provincial, regional and national roads where the cyclist’ maneuver was unknown or (1) at day time, or (2) when the pavement was dry, or (3) the cyclists hit a stopped vehicle. Association rules also showed that male cyclists riding through crossroads had an increased probability of having a fatal bicycle accident. Association rules for C13 highlighted that cyclists that were 65 or more years old and riding during spring were more likely to incur in a fatal bicycle accident. Furthermore, association rules showed an increased likelihood of fatal bicycle crashes when the type of collision was a head-on and it (1) took place during weekdays, or (2) occurred at crossroads, or (3) happened when weather was clear, or (4) involved an opponent vehicle which was not keeping a safe distance. Association rules for C14 indicated that fatal bicycle accidents were more probable when the opponent vehicle was a truck and (1) the collision occurred during daytime, or (2) happened when weather was clear, or (3) the collision type was side-impact, and or (4) the cyclist’ s gender was male. Association rules also showed an increased probability of fatal bicycle accident when the collision type was a head-on and the cyclist was riding straight forward. Association rules for C15 showed that fatal bicycle accidents were more likely to occur when the opponent vehicle was a truck and (1) the cyclist was riding straight forward, or (2) there were both vertical and horizontal road signs, or (2) the weather was clear, or (3) during weekdays, or (4) when the pavement was dry. Association rules for C16 indicated that fatal bicycle accidents were more likely to occur when the opponent vehicle was a truck and occurred on rural roads. Furthermore, fatal bicycle accidents were more likely to occur if the cyclist was 65 or more years old and (1) the cyclist’s gender was male, or (2) the cyclist was not at a junction or a roundabout, or (3) during weekdays. Association rules for C17 showed that fatal bicycle accidents were more probable when the cyclist was 65 or more years old and (1) was autumn, or (2) the type of collision was a rear-end. Association rules also showed that fatal bicycle crashes were most likely to occur during the weekend and in autumn; on rural roads, with a rear-end type of collision; and where the road signage was absent and the cyclist was not driving at a junction or a roundabout. Association rules for C18 showed that fatal bicycle accidents were more likely to occur or rural roads. Rules also showed that the probability of a fatal bicycle crashes increased when the cyclist was 65 or more years old and (1) the cyclist’s gender was male, or (2) the cyclist’s maneuver was unknown, or (3) the cyclist was riding during spring, or (4) the road signage was both vertical and horizontal. Association rules for C19 indicate that fatal bicycle accidents were probable on rural roads. They were also probable when the weather was clear (1) during the weekend or (2) the cyclist was not respecting the right of way. Furthermore, association rules showed that fatal bicycle crashes were more likely when the cyclist was 65 or more years old and (1) the cyclist was not respecting the right of way, or (2) when the cyclist’s gender was male.

## Discussion

In this article, we examined bicycle crashes in Italy using cluster analysis and data mining techniques. Specifically, the present study revealed 19 latent classes of distinguishable bicycle crash patterns. These 19 latent classes were different in terms of features of the bicycle crashes. In addition, logistic regression revealed that the likelihood of fatal injury was different among these 19 classes and association rules uncovered different circumstances associated with the bicycle crashes among the classes. In line with a few previous studies that used traffic accident data segmentation techniques [29, 61–65], we demonstrated that clustering techniques can be very useful in finding various subsets of crashes in a heterogeneous traffic accident data set. The 19 latent classes represent different intelligible and justifiable bicycle crash types, which uncover information that remains hidden due to data heterogeneity. Moreover, logistic regression analysis revealed that the 19 classes differ in terms of their injury severity levels, meaning that some crash typologies were characterized by relatively low or high cyclists’ fatal injury severity levels.

The discussion section is structured as follows. First, we discuss the different classes identified and describe their main characteristics and causal paths potentially present. Second, we address the factors that increase the likelihood of accident in each class, and discuss the role of gender and age in crashes in general, given the lack of influence on the classes. Finally, we talk about the implications of the findings and the limitations of the study.

In the following paragraphs, we first describe the main characteristics that accident classes have in common, we then analyze how accident classes differ from each other and the implications for the underlying causation.

**Fig 4:**
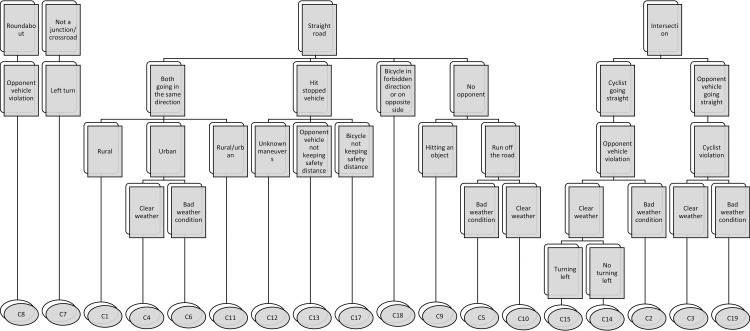
Structure of the 19 classes based on their prevalent characteristics.

Fig 4 displays the structure of the 19 classes based on their prevalent characteristics. As Fig 4 shows, the 19 classes bear similarities among them regarding several dimensions (e.g., presence of intersection, type of manoeuver). This way, a broad distinction can be made based on the infrastructure layout (i.e., roundabout, intersection, straight road). Crashes taking place at roundabout were mainly represented by C8, which also mainly feature violations by opponent vehicle drivers. Accidents chiefly taking place at intersection span five classes, which can be differentiated according to road users’ manoeuvers. Those accidents in which the cyclist or motorist was riding straight forward, the opponent vehicle was committing a violation, and there was bad weather are represented by C2 or C19. Whereas those with clear weather conditions can be split into those in which the opponent vehicle was turning left (i.e., C15) or not doing so (i.e., C14).

Those accidents taking place on a straight road are represented by 11 classes. Among these, accidents in which the cyclist was riding on the opposite direction or side are represented by class 18. Accidents without an opponent vehicle can be split between those in which cyclists hit an object (i.e., C9), and those in which they ran off the road, both with bad weather (i.e., C5) and clear one (i.e., C10). Accidents in which cyclists hit a stopped vehicle are divided into those in which the safety distance was not respected by cyclists (i.e., C17), by another vehicle (i.e., C13), or those with unknown manoeuver (i.e., C12). Accidents in which both cyclists and opponent vehicle travelled in the same direction are divided into those befalling on rural roads (i.e., C1), in urban roads with clear (i.e., C4) and bad weather (i.e., C6), and C11 for those that did not fall exactly under the other classes. Finally, accidents not taking place either at junction or crossroad, and involving a left turn, are represented by C7.

Nevertheless, different causal sequences may underlie diverse crash characteristics. C2, C3, C14, C15, and C19 involve the presence of an intersection, which might entail specific challenges for drivers regarding awareness and cyclists’ visibility and comprehension. This could be addressed by using specific advanced driver assistance systems comprising cautionary collision warnings and information about the intersection [66]. Nevertheless, they differ in several aspects. In C2, C14 and C15, the most common cyclist’s maneuver was to ride straightforward, whether the opponent vehicle was committing a violation. Yet, bicycle crashes corresponding to C2 typically happened in winter or autumn during rain when the opponent vehicle was not respecting the right of way. The bad weather condition seems to indicate that such collisions could be due to low visibility of either the cyclist or the road signs and the reduction of visual search strategies associated with such environmental conditions [67]. Moreover, given the rain, cyclists might be slightly rushed to arrive to their destination as soon as possible and, therefore, probably being less careful at intersections assuming their right to pass and not foreseeing that the opponent vehicle couldn’t detect them. On the other hand, bicycle crashes in C14 are mainly characterized by the same features, nevertheless, the most of them took place during spring or summer, the weather was clear the most of the times and there was a higher percentage of opponent vehicles ignoring a red light. This might lead to discarding low visibility due to weather conditions, while possibly attributing it to some visual obstruction at the intersection or to the intersection structural configuration itself. Moreover, the fact that the percentage of red light skipping is higher implies a higher probability of violations accounting for the maneuvers leading to bicycle crashes. Finally, bicycle crashes that belong to C15 involved the same type of maneuvers by the opponent vehicle, but also included the left turn. Moreover, the majority of bicycle crashes took place during spring, autumn and summer, and more than a 40% of the cyclists were women. These bicycle crashes as well as those of C14 might be due to cyclist’s greater exposure and probably higher speeds (and riskier behaviors) by both cyclists and opponent vehicles [68] in those seasons. Yet, the fact that the bicycle crashes are more frequent during seasons other than winter might be related to a higher number of cyclists on the road during those seasons. Several studies already highlighted that during seasons characterized by warmer temperature and less rainfall the volume of cyclists increases [69–71].

Regarding bicycle crashes included in C3 and C19, they have in common that the opponent vehicle’s maneuver was typically going straightforward, while the cyclist was clearly committing a violation or an error (i.e., skipping red-lights or not respecting the stop sign). Nevertheless, bicycle crashes included in C3 mostly took place during spring or summer when there was clear weather, whereas those in C19 involved bicycle crashes during winter or autumn when it was raining. As well as for the foregoing classes, the bicycle crashes taking place during bad weather conditions (i.e., C19) could be due to low visibility of the intersection signs. Furthermore, given that the cyclist is typically the one who violates in this class, the low visibility of the opponent vehicles could be a factor that increases the probability of red-light skipping by the cyclist [72]. There is evidence that the failure to detect motorized vehicles was an important factor in explaining bicycle crashes [73]. Such violations or errors might also be due to the cyclist rushing toward the destination to avoid rain [72]. As per the bicycle crashes that took place under clear weather, two possible causes could be (1) a cyclist’s failure to detect the incoming vehicles (i.e., looked but failed to see), or (2) a cyclist’s misjudgment of the time and speed to cross without being caught by the opponent vehicle, or in other words, a misjudgment of the safe gap in the traffic stream. In fact, cyclists may violate traffic red-light if the gap in the conflicting stream is big enough to avoid a possible collision and, according to Van der Meel (72), this evaluation can be misled by a variety of factors and elements on the road (e.g., crossing distance, clarity of the intersection, intersection design, composition and velocity of the conflicting traffic).

C1, C4, C6, and C11 comprise bicycle crashes that did not take place at junction and involved both the cyclists and the opponent vehicle being in motion and going in the same direction. The main differences are analyzed next. Bicycle crashes comprised in C1 mostly took place on rural roads, more than 20% were against trucks or multiple vehicles, and the opponent vehicle was driving straightforward while the cyclist typically performed an unknown maneuver, which most probably stands for distraction. The side and rear end impact in combination with the unknown maneuver and the type of location not at junction leads to think of a possible overtaking attempt by the opponent vehicle without respecting the safety distance when the cyclist was not riding totally straightforward, either due to an eventual encounter of an obstacle, distractions or the narrowness of the hard shoulder. Another possible explanation would be that of low visibility due to a curve [74] or a change in the gradient of the road, in combination with a lack of expectation to encounter a cyclist by the driver of the opponent vehicle (because of the rural context). The rest of the classes feature bicycle crashes that took place mostly on urban roads, in which higher traffic volume can be expected. In bicycle crashes comprised in C4, the cyclist was typically riding straightforward whereas the opponent vehicle’s maneuver was unknown, differently from bicycle crashes included in C1. Among the potential explanations for this accident pattern are higher mixed traffic intensity, meaning that (1) there will be more vehicles merging into lanes, possibly without detecting the cyclist or (2) drivers could overtake the cyclists leaving less space due to the higher number of vehicles incoming in the opposite lane; and (3) the driver’s distraction that would be classified under the label “unknown” (e.g., phone use). In C6, a higher percentage of cyclist’s maneuvers was unknown (i.e., 46.9%) and the most of them took place during winter or autumn when rain was present (whereas for the rest of the classes, the weather was clear). Possible causes for this pattern are slippery road due to bad weather, vehicles merging into a lane without detecting bicyclists due to low visibility, or not expecting cyclists due to the season and especially due to weather conditions. Finally, those in C11 involved opponent vehicles not respecting the safety distance or speeding and the collision was on the rear end. Moreover, they mostly took place during summer or spring. A possible explanation would be the driver misjudgment of the speed or distance to the cyclist ahead.

Accidents mainly involving hitting a stopped vehicle not being at junction (i.e., C12, C13, and C17), take place mostly during spring and summer when the weather was clear. Manoeuvers classified as unknown also comprise distraction, which should be taken into consideration when analyzing the potential causes of each patter. In C12, which mainly features accidents with unknown manoeuvers by both road users, bicycle crashes could be due to distraction of both the cyclist and the opponent vehicle driver. Whereas those in C13, in which the opponent vehicle did not keep the safety distance, and C17, where bicyclists were those not keeping the safety distance, might involve road users’ distraction (e.g., smartphone devices) or overestimating the distance to the vehicle ahead and not being able to avoid the collision when it stopped.

The characteristics of C7 deserve separate consideration. To begin with, it is the only cyclist-motorist crash pattern in which the cyclist was turning left in most of the cases (i.e., 77%). The bicycle crashes occurred during daytime in spring and summer, when the weather was clear and the urban road surface was dry. Another interesting aspect is that, in this cluster, bicycle crashes could occur in the presence of a crossroad (44.9%) or not (52.5%). This highlights the riskiness of the turning left maneuver for cyclists. The most frequent maneuver of the opponent vehicle was driving straightforward, and just in few cases it was overtaking (11.7%) or traveling too fast (10.0%). These cluster characteristics could entail a misjudgment of the opponent vehicle’s speed by the cyclist [73]. In this case, while turning left to eventually stop on the other side of the road, a cyclist could misjudge the best time to cross the road, thus, being hit by the oncoming vehicle. The cyclist-motorist crash pattern here described could also be due to the cyclist that did not properly signal the turning left maneuver, thus, misleading the driver, who is not ready to react in time (e.g., because of traveling too fast or having already begun the overtaking maneuver).

Despite the fact that in most of the cases the cyclist’s maneuver was unknown, the accident in C18 involved a cyclist riding in a forbidden direction or on opposite side of the road (20% of the cases). The bicycle crashes occurred on a straight road, during daytime, in spring and summer, when the weather was clear. In these cases, it could be that the driver of the opponent vehicle was not expecting any oncoming road user, leading to a side-impact. Another possible explanation could be that in urban context, one-way roads are generally narrower and counterflow riding by the cyclists could lead to a side impact when encountering oncoming motorized vehicles, especially if there are obstacles on the road. It could be helpful to explore why a cyclist decides to ride on an opposite side of the road or in a forbidden direction to prevent those types of bicycle crash. For example, a cyclist could decide to ride on a forbidden direction or on an opposite side of the road because (1) it could reduce the time needed to get to desired destination or (2) it could be perceived safer than other more congested and busier roads.

C8 is the only cluster that features collisions between bicycles and motorized vehicles typically happening at roundabouts. The bicycle crashes took place chiefly during daytime in spring and summer, when the weather was clear and the urban road surface was dry. In this cyclist-motorist crash pattern, the cyclists were riding straight forward (88.9%) and the drivers did not respect the right of way (71.0%). This could entail the presence of a temporary obstruction to the field of view [66] or a looked but failed to see episode [75–77]. Research literature often stresses how likely happens that a driver is sure to have the road free when approaching a road while, instead, a cyclist is coming. The looked but failed to see episodes often happen because of a poor scanning strategy by the driver who is paying more attention to other car drivers, and completely overlooks the presence of a cyclist [66, 75–77]. Another possible cause could be the drivers’ negative attitude toward cyclists [78] which was seen to influence the driving behavior. In particular, drivers holding negative attitudes toward cyclists were associated with poorer knowledge of road rules (i.e., not respecting the right of way) which could also explain bicycle crashes included in C8.

Regarding the classes featuring accidents without opponent vehicles (i.e., C5, C9, and C10), they differ in several conditions, which are detailed next. To begin with, most of the bicycle crashes belonging to C5 took place in rainy conditions, involved an unknown maneuver and the main outcomes were going off the road or unspecified. The main difference of C5 with patterns in C9 and C10 was the bad weather condition, which can be attributed as a cause of this type of accident. In fact, bad weather can lead to lower visibility of obstacles on the road, slip due to the wet pavement and lose control of the bike, underestimating the severity of the pavement conditions, or overestimating the own capacity to have control of the bicycle. Bicycle crashes included in C9 typically took place under clear weather conditions and involved hitting an object while riding straightforward, which implies that it could be due to a door of a parked vehicle that suddenly opens (“dooring” crashes), hitting a pedestrian or colliding with some unexpected object. Those belonging to C10 bear a strong resemblance with those in C5, but do not take place during bad weather conditions. Given the absence of an opponent vehicle and the high percentage of unknown maneuvers in C5 and C10, all the accident patterns comprised in them might eventually involve (1) bicycle mechanical conditions, (2) cyclist’s intoxication, (3) cyclist’s distraction leading to a collision against undetected objects, (4) cyclist’s errors due to lack of experience (i.e., occasional cyclist).

Finally, C16 encompasses bicycle crashes that mostly took place at crossroads or not at junction and with mostly unknown maneuvers for both the cyclist and the opponent vehicle. This leads to thinking that this might be a hodgepodge of events that could not be assigned to other classes because the maneuvers were unknown. What makes us think so is the fact that there is no prevalent location of the road in which bicycle crashes might typically happen. Nonetheless, the fact that the maneuvers are unknown could be due to a real lack of knowledge of what happened when registering the collision by the competent authority, or to the absence of a category in the classification of maneuvers used to code the accident.

What is more, having a truck as an opponent vehicle entailed a higher risk of fatality for classes mainly associated with crossroads (i.e., C14 and C15) and roundabout (C8). As previous research shows [17, 47], collisions with trucks entail a higher probability of fatality injury severity. The increased risk of fatality associated with such heavier vehicles could be expected due to their higher momentum [47]. Nevertheless, a closer look at the exact characteristics of the bicycle crashes that could better explain the scenario and the relationship with the momentum is needed. The majority of the collisions in C8, C14, and C15 involve cyclists riding straightforward and opponent vehicles committing a violation (e.g., not respecting the right of way) leading to a side-impact. This side-impact could translate into heavier vehicles hitting a wider surface of the cyclist and transmitting more energy (as opposed to both vehicles sharing a common trajectory), therefore leading to more severe injuries, which is consistent with previous findings [17].

Another finding of association rules revealed the dangerousness of riding on a rural road. In line with previous research [17–20], a bicycle crash on rural road was more likely to be fatal compared to urban road. One intuitive explanation could be the higher posted speed limit of rural road, which increases safety risk for all road users, including cyclists. Previous research showed the role of speed as a factor for bicycle-motor vehicle collision resulting in a more severe crash due to a high differential in operating speeds and an even greater impact when compared to an urban cyclist-motorist crash [17, 18, 79, 80]. Furthermore, assuming that bicycle usage is typically higher on urban than rural roads [79, 81], we can also suppose that a car driver is not expecting a cyclist riding on this type of road, which could lead to an unfortunate collision due to a lack of capacity to react and avoid the crash.

The findings of the present study suggest that gender and age of cyclists were not important in determining the features of the latent classes. In other words, cyclists of different gender and age were involved in the different subgroups of bicycle crashes with small or negligible differences. Consistent with the finding that male cyclists are more likely to be involved in crashes than female cyclists [33, 44], our study found that the percentages of male cyclists exceeded those of female cyclists in all 19 latent classes. It should be noted that this finding does not necessarily mean that female and male cyclists have a different risk of having a crash. The most likely explanation is related to exposure: male cyclists tend to cycle more frequently, during a longer time and greater distances compared to female cyclists [30]. Based on the findings of the present study and those of previous reports [30, 53], we conclude that the gender of cyclists is not likely to play an important role in the likelihood of being involved in bicycle crashes when controlling for exposure. Nevertheless, we did find some small gender differences. Female cyclists were notably less likely to be involved in C1 and C14. Based on odds ratio values from logistic regression, C1 is the most dangerous type of bicycle crashes. In addition, the findings of association rules suggest that male cyclists are more likely to suffer fatal injuries compared to female cyclists. Both results from latent class analysis and association rules provide some support to the notion that male cyclists were more likely to be involved in fatal bicycle crashes than female cyclists [19, 26, 50].

A cyclist’s age does not seem to play an important role in the likelihood of being involved in the 19 types of bicycle crashes too. We note that among younger cyclists (0–14 and 15–24 years) the percentages of cluster observations were notably higher in C3 and C18. A violation committed by the cyclist (i.e., ignoring stop signs or red light traffic/not respecting the right of way/driving in a forbidden direction or on an opposite side of the road) is one of the major features of the bicycle crashes included in these subgroups. This finding could be explained by the inexperience of young cyclists as road users [82] and suggest the need to implement effective road safety education programs for young cyclists [83]. In addition, findings of association rules revealed that cyclists aged 65 years or more were more likely to be involved in fatal bicycle crashes than younger cyclists. This finding is in line with the results of previous reports that showed a higher risk of injury severity as age increases [18, 23, 26, 28, 33, 41, 45–49]. In this respect, two explanations have been advanced: physical fragility (susceptibility to injury) and crash over-involvement due to of unsafe driving [45, 84, 85]. The finding that among cyclists aged 65 years or older the percentage of cluster observations was notably higher in C7 (where the maneuver of the cyclist is turning left and findings of logistic regression suggest that this type of crash is dangerous) may suggest that unsafe driving may play a role, albeit limited, in explaining the higher risk of injury severity. However, based on the findings of both cluster analysis and association rules, we can conclude that the increased likelihood of sustaining a fatal injury was more likely to be due to susceptibility to injury due to fragility.

### Implications for practice

Although the 19 classes of bicycle crashes share the some of the features, different causes and profiles of road users can be identified, and therefore, they should be addressed differently when it comes to prevention. To prevent bicycle crashes comprised in C2, more visible signs [86] at intersections are needed and cyclists’ visibility should be increased by using flashing lights, lamps, and retro-reflective materials that work better for night-time visibility [87]. Nevertheless, introducing visibility measures to increase safety could eventually lead to the negative risk compensation phenomenon [88], therefore, it would be advisable to also address the visibility issue both by educational campaigns and implementation of more conspicuous signs [86, 89]. To prevent the bicycle crashes comprised in C14 and C15, a more reasonable approach could consist in road safety advertisement campaigns [90] to increase awareness of cyclists and their vulnerability.

A way to address traffic red light violations in C3 and C19 could be by shortening red light waiting times to maximize compliance, as suggested by previous studies on pedestrians [91], or otherwise improving the intersection designs to maximize clarity and increasing speed traffic calming measures. In addition, to address violations due to the cyclist rushing toward the destination to avoid rain (i.e., C19), a fruitful approach may be the installation of rain sensors that communicate and allow for shortening red light waiting time or increasing the green one for cyclists during such weather conditions.

To address misperception and misjudgment of speed and distance to the bicyclist ahead by the driver of the opponent vehicle (i.e., in C1, C4, C6, C8, C11, C12, and C13), there if need for a stronger focus on the infrastructure (i.e., segregation of traffic by building cycle tracks) and warning about the possible presence of cyclists at curves and changes of gradient might be needed [92].

Finally, violations and errors characterize most of the classes of bicycle crashes (e.g., C2, C3, C5, C8, C9, C10, C13, C14, C15, C17, C18, and C19). Therefore, comprehensive traffic education and training of both cyclists and motorists is recommended to address these violations and errors. On the one hand, it seems useful a stringent training of motorists to respect cyclists and avoid hitting them. On the other hand, extensive training in safe and effective cycling techniques could promote safe and convenient cycling [93].

### Limitations

Some limitations should be kept in mind when interpreting and generalizing the findings of this study. The findings of this study are dependent on the way that data have been collected. First of all, the nature of the study itself, analyzing only data of traffic crashes happened in Italy, does not permit to generalize the results and the implications to other countries. Secondly, even though surpassing the speed limit can be determined objectively once an estimation of pre-crash speed is obtained, the article 141 of the Italian Highway Code specifies that under certain conditions (e.g., low visibility, nearby schools, or places that children frequent, narrow streets) vehicles and other road users (e.g., bicycles) are supposed to slow down. In the absence of such speed reduction, road users are thought to go excessively fast and can be fined according to the aforementioned law. Nonetheless, the threshold from which a given speed is deemed excessive under one of those given circumstances is not determined by the code. This could lead to different interpretations by police officers and, therefore, it constitutes a limitation of the data collection process. Moreover, different findings might have been obtained if other types of variables (e.g., speed limits or alcohol use) had been collected. We note that ISTAT database is currently the most comprehensive collection of road crashes in Italy. Regarding some of the specific variables considered, in particular opponent vehicle’s and cyclist’s maneuver the sub-category “unknown or other” accounts for a high percentage. This is due to the fact that in those cases it was not possible to ascertain the pre-crash maneuver with any certainty. Moreover, to our knowledge, no study has assessed the reliability of the Italian official road accidents data. Data are collected by the authority that dealt with the event, such as Traffic Police, Carabinieri, Provincial or Municipal Police. Nevertheless, the fact that police officers can be prosecuted if they make a false statement and that information regarding the details of the accident, its causes or relevant circumstances can be used in court trials constitutes a proxy for its accuracy and reliability. Another limitation regards the results obtained in this study which are dependent on analyses used. In latent class analysis, the found solution might be a local maximum likelihood of the latent class model, rather than a global maximum. To ensure that the global maximum likelihood of the latent class model has been achieved, we estimated each class model ten times and save the one model of the ten that had the greatest likelihood. Another consideration relates to the selection of the parameters in association rules. The use of different parameters can lead to different results. In the present article, we decided to consider the results with higher confidence and to select those that had a minimum support value of 5% as it was done in line with a previous study [60].

### Conclusion

In this paper, we analyzed and classified the bicycle crashes that took place in Italy from 2011 to 2013. We employed latent class analysis using categorical indicators related to infrastructure, road user, vehicle, and environmental/time characteristics to perform the classification of the bicycle crashes. The 19 classes of bicycle crashes have different causes, degree of severity, and profiles of road users. The classes distinguished cluster of patterns that involved bicycle crashes that did take place at intersection, not at intersection, and at roundabout. Within the classes of bicycle crashes that took place at intersection, we have examined those that tend to involve a car driver’s violation and a cyclist’s violation. Among the classes of bicycle crashes that took place in a straight road, patterns entailed hitting a stopped vehicle, bicycle riding in forbidden direction or on the opposite side of the road, bicycle and opponent vehicle going in the same direction, and no opponent vehicle. Separate consideration was given to the classes involving the left turn scenario and bicycle crashes that took place at a roundabout. In addition, the application of logistic regression and association rules allowed to identify different conditions for the latent classes that entail a higher risk of fatality. Specifically, the involvement of male cyclists or cyclists aged 65 years or older, having a truck as an opponent vehicle, and riding on a rural road entailed a higher risk of fatality. We argue that the design and implement the most effective countermeasures should be tailored to the specific type of bicycle crash.

## Supporting information

S1 Table Confusion Matrix. (DOCX)

S2 Table Latent Class Characteristics (Percentage of Cluster Observations). (DOCX)

S3 Table Rules with Bicycle Fatalities as Consequent. (DOCX)

## References

[1] European Commission. Quality of transport. Special Eurobarometer 422a / Wave EB82.2 –TNS Opinion & Social. 2014.

[2] Kelly P, Kahlmeier S, Gotschi T, Orsini N, Richards J, Roberts N, et al Systematic review and meta-analysis of reduction in all-cause mortality from walking and cycling and shape of dose response relationship. Int J Behav Nutr Phys Act. 2014;11:132 Epub 2014/10/26. PubMed Central PMCID: PMCPmc4262114. 10.1186/s12966-014-0132-x 25344355 PMC4262114

[3] Götschi T, Garrard J, Giles-Corti B. Cycling as a part of daily life: A review of health perspectives. Transport Reviews. 2016;36(1):45–71.

[4] Taddei C, Gnesotto R, Forni S, Bonaccorsi G, Vannucci A, Garofalo G. Cycling promotion and non-communicable disease prevention: health impact assessment and economic evaluation of cycling to work or school in Florence. PLoS ONE. 2015;10(4):e0125491 10.1371/journal.pone.0125491 25928421 PMC4415918

[5] Rojas-Rueda D, de Nazelle A, Andersen ZJ, Braun-Fahrländer C, Bruha J, Bruhova-Foltynova H, et al Health impacts of active transportation in Europe. PLoS ONE. 2016;11(3):e0149990 10.1371/journal.pone.0149990 26930213 PMC4773008

[6] Wojan TR, Hamrick KS. Can walking or biking to work really make a difference? Compact development, observed commuter choice and body mass index. PLoS ONE. 2015;10(7):e0130903 10.1371/journal.pone.0130903 26154176 PMC4495983

[7] de Nazelle A, Nieuwenhuijsen MJ, Anto JM, Brauer M, Briggs D, Braun-Fahrlander C, et al Improving health through policies that promote active travel: A review of evidence to support integrated health impact assessment. Environ Int. 2011;37(4):766–77. Epub 2011/03/23. 10.1016/j.envint.2011.02.003 21419493

[8] Xia T, Zhang Y, Crabb S, Shah P. Cobenefits of replacing car trips with alternative transportation: A review of evidence and methodological issues. J Environ Public Health. 2013;2013:14. 10.1155/2013/797312 PMC3730154 23956758

[9] Macmillan A, Connor J, Witten K, Kearns R, Rees D, Woodward A. The societal costs and benefits of commuter bicycling: Simulating the effects of specific policies using system dynamics modeling. Environ Health Perspect. 2014;122(4):335–44. Epub 2014/02/06. PubMed Central PMCID: PMCPmc3984216. 10.1289/ehp.1307250 24496244 PMC3984216

[10] Heinen E, van Wee B, Maat K. Commuting by bicycle: An overview of the literature. Transport Reviews. 2010;30(1):59–96.

[11] Schepers P, Hagenzieker M, Methorst R, van Wee B, Wegman F. A conceptual framework for road safety and mobility applied to cycling safety. Accid Anal Prev. 2014;62:331–40. 10.1016/j.aap.2013.03.032 23623174

[12] Kaplan S, Giacomo Prato C. A spatial analysis of land use and network effects on frequency and severity of cyclist–motorist crashes in the Copenhagen region. Traffic Inj Prev. 2015;16(7):724–31. 10.1080/15389588.2014.1003818 25664847

[13] Dumbaugh E, Li W. Designing for the Safety of Pedestrians, Cyclists, and Motorists in Urban Environments. J Am Plann Assoc. 2010;77(1):69–88.

[14] Liu X, Shen D, Huang J. Analysis of bicycle accidents and recommended countermeasures in Beijing, China. Transportation research record. 1995;(1487):75–83.

[15] Minikel E. Cyclist safety on bicycle boulevards and parallel arterial routes in Berkeley, California. Accid Anal Prev. 2012;45:241–7. 10.1016/j.aap.2011.07.009 22269506

[16] Wessels R. Bicycle collisions in Washington State: A six-year perspective, 1988–1993. Transportation Research Record: Journal of the Transportation Research Board. 1996;1538:81–90.

[17] Moore DN, Schneider IV WH, Savolainen PT, Farzaneh M. Mixed logit analysis of bicyclist injury severity resulting from motor vehicle crashes at intersection and non-intersection locations. Accid Anal Prev. 2011;43(3):621–30. 10.1016/j.aap.2010.09.015 21376847

[18] Boufous S, de Rome L, Senserrick T, Ivers R. Risk factors for severe injury in cyclists involved in traffic crashes in Victoria, Australia. Accid Anal Prev. 2012;49:404–9. 10.1016/j.aap.2012.03.011 23036419

[19] Amoros E, Chiron M, Thélot B, Laumon B. The injury epidemiology of cyclists based on a road trauma registry. BMC Public Health. 2011;11(1):1–12. 21849071 10.1186/1471-2458-11-653 PMC3176219

[20] Macpherson AK, To TM, Parkin PC, Moldofsky B, Wright JG, Chipman ML, et al Urban/rural variation in children’s bicycle-related injuries. Accid Anal Prev. 2004;36(4):649–54. 10.1016/S0001-4575(03)00086-1 15094419

[21] Chen L, Chen C, Srinivasan R, McKnight CE, Ewing R, Roe M. Evaluating the safety effects of bicycle lanes in New York City. Am J Public Health. 2012;102(6):1120–7. 10.2105/AJPH.2011.300319 22095351 PMC3483943

[22] Romanow NTR, Couperthwaite AB, McCormack GR, Nettel-Aguirre A, Rowe BH, Hagel BE. Environmental determinants of bicycling injuries in Alberta, Canada. J Environ Public Health. 2012;2012:12. 10.1155/2012/487681 PMC3515916 23251192

[23] Stone M, Broughton J. Getting off your bike: Cycling accidents in Great Britain in 1990–1999. Accid Anal Prev. 2003;35(4):549–56. 12729818 10.1016/s0001-4575(02)00032-5

[24] Wei F, Lovegrove G. An empirical tool to evaluate the safety of cyclists: Community based, macro-level collision prediction models using negative binomial regression. Accid Anal Prev. 2013;61:129–37. 10.1016/j.aap.2012.05.018 22721549

[25] Vandenbulcke G, Thomas I, Int Panis L. Predicting cycling accident risk in Brussels: A spatial case–control approach. Accid Anal Prev. 2014;62:341–57. 10.1016/j.aap.2013.07.001 23962661

[26] Bíl M, Bílová M, Müller I. Critical factors in fatal collisions of adult cyclists with automobiles. Accid Anal Prev. 2010;42(6):1632–6. 10.1016/j.aap.2010.04.001 20728611

[27] Klop J, Khattak A. Factors influencing bicycle crash severity on two-lane, undivided roadways in North Carolina. Transportation Research Record: Journal of the Transportation Research Board. 1999;1674:78–85.

[28] Eluru N, Bhat CR, Hensher DA. A mixed generalized ordered response model for examining pedestrian and bicyclist injury severity level in traffic crashes. Accid Anal Prev. 2008;40(3):1033–54. 10.1016/j.aap.2007.11.010 18460372

[29] Kaplan S, Giacomo Prato C. Cyclist-motorist crash patterns in Denmark: A latent class clustering approach. Traffic Inj Prev. 2013;14(7):725–33. Epub 2013/08/16. 10.1080/15389588.2012.759654 23944832

[30] de Geus B, Vandenbulcke G, Int Panis L, Thomas I, Degraeuwe B, Cumps E, et al A prospective cohort study on minor accidents involving commuter cyclists in Belgium. Accid Anal Prev. 2012;45:683–93. 10.1016/j.aap.2011.09.045 22269558

[31] Gerberich SG, Parker D, Dudzik M. Bicycle-motor vehicle collisions. Epidemiology of related injury incidence and consequences. Minn Med. 1994;77(4):27–31. Epub 1994/04/01. 8007909

[32] Nicaj L, Stayton C, Mandel-Ricci J, McCarthy P, Grasso K, Woloch D, et al Bicyclist Fatalities in New York City: 1996–2005. Traffic Inj Prev. 2009;10(2):157–61. 10.1080/15389580802641761 19333828

[33] Yan X, Ma M, Huang H, Abdel-Aty M, Wu C. Motor vehicle–bicycle crashes in Beijing: Irregular maneuvers, crash patterns, and injury severity. Accid Anal Prev. 2011;43(5):1751–8. 10.1016/j.aap.2011.04.006 21658503

[34] Ashbaugh SJ, Macknin ML, VanderBrug Medendorp S. The Ohio bicycle injury study. Clin Pediatr (Phila). 1995;34(5):256–60. 7628167 10.1177/000992289503400505

[35] Hamann JC, Peek-Asa C, Lynch CF, Ramirez M, Hanley P. Epidemiology and spatial examination of bicycle-motor vehicle crashes in Iowa, 2001–2011. Journal of Transport & Health. 2015;2(2):178–88. 10.1016/j.jth.2014.08.006 PMC9348608 35928557

[36] Hunter WW, Pein WE, Stutts JC. Bicycle-motor vehicel crash types: The early 1990s. Transportation Research Record. 1995;(1502):65–74.

[37] Vandenbulcke G, Thomas I, de Geus B, Degraeuwe B, Torfs R, Meeusen R, et al Mapping bicycle use and the risk of accidents for commuters who cycle to work in Belgium. Transp Policy. 2009;16(2):77–87.

[38] Silvano AP, Koutsopoulos HN, Ma X. Analysis of vehicle-bicycle interactions at unsignalized crossings: A probabilistic approach and application. Accid Anal Prev. 2016;97:38–48. 10.1016/j.aap.2016.08.016 27565043

[39] Stimpson JP, Wilson FA, Muelleman RL. Fatalities of pedestrians, bicycle riders, and motorists due to distracted driving motor vehicle crashes in the U.S., 2005–2010. Public Health Rep. 2013;128(6):436–42. Epub 2013/11/02. PubMed Central PMCID: PMCPMC3804087. 24179255 10.1177/003335491312800603 PMC3804087

[40] Garder P. Bicycle accidents in Maine: An analysis. Transportation Research Record. 1994;1438:34–41.

[41] Eilert-Petersson E, Schelp L. An epidemiological study of bicycle-related injuries. Accid Anal Prev. 1997;29(3):363–72. 9183474 10.1016/s0001-4575(97)00002-x

[42] Sayed T, Zaki MH, Autey J. Automated safety diagnosis of vehicle–bicycle interactions using computer vision analysis. Saf Sci. 2013;59:163–72.

[43] Wang Y, Nihan NL. Estimating the risk of collisions between bicycles and motor vehicles at signalized intersections. Accid Anal Prev. 2004;36(3):313–21. 10.1016/S0001-4575(03)00009-5 15003575

[44] Tin Tin S, Woodward A, Ameratunga S. Injuries to pedal cyclists on New Zealand roads, 1988–2007. BMC Public Health. 2010;10. 10.1186/1471-2458-10-655 PMC2989960 21034490

[45] Schepers P. Does more cycling also reduce the risk of single-bicycle crashes? Inj Prev. 2012;18(4):240–5. 10.1136/injuryprev-2011-040097 22109243

[46] Rivara FP, Thompson DC, Thompson RS. Epidemiology of bicycle injuries and risk factors for serious injury. Inj Prev. 2015;21(1):47–51. 10.1136/injprev-00002-0038rep 25609752

[47] Kim J-K, Kim S, Ulfarsson GF, Porrello LA. Bicyclist injury severities in bicycle–motor vehicle accidents. Accid Anal Prev. 2007;39(2):238–51. 10.1016/j.aap.2006.07.002 17005154

[48] Rodgers GB. Factors associated with the crash risk of adult bicyclists. J Safety Res. 1997;28(4):233–41.

[49] Ekman R, Welander G, Svanstrom L, Schelp L, Santesson P. Bicycle-related injuries among the elderly—a new epidemic? Public Health. 2001;115(1):38–43. Epub 2001/06/13. 10.1038/sj.ph.1900713 11402350

[50] Haileyesus T, Annest JL, Dellinger AM. Cyclists injured while sharing the road with motor vehicles. Inj Prev. 2007;13(3):202–6. 10.1136/ip.2006.014019 17567979 PMC2598373

[51] Ackery AD, McLellan BA, Redelmeier DA. Bicyclist deaths and striking vehicles in the USA. Inj Prev. 2012;18(1):22–6. 10.1136/injuryprev-2011-040066 21890578

[52] McCarthy M, Gilbert K. Cyclist road deaths in London 1985–1992: Drivers, vehicles, manoeuvres and injuries. Accid Anal Prev. 1996;28(2):275–9. 8703286 10.1016/0001-4575(95)00061-5

[53] Hoffman MR, Lambert WE, Peck EG, Mayberry JC. Bicycle commuter injury prevention: It is time to focus on the environment. J Trauma. 2010;69(5):1112–7; discussion 7–9. Epub 2010/11/12. 10.1097/TA.0b013e3181f990a1 21068616

[54] Brand S, Otte D, Petri M, Müller C, Stübig T, Krettek C, et al Bicyclist–bicyclist crashes—A medical and technical crash analysis. Traffic Inj Prev. 2013;14(1):56–60. 10.1080/15389588.2012.688152 23259519

[55] Wang C, Lu L, Lu J. Statistical analysis of bicyclists’ injury severity at unsignalized intersections. Traffic Inj Prev. 2015;16(5):507–12. 10.1080/15389588.2014.969802 25310346

[56] Klassen J, El-Basyouny K, Islam MT. Analyzing the severity of bicycle-motor vehicle collision using spatial mixed logit models: A City of Edmonton case study. Saf Sci. 2014;62:295–304.

[57] R Core Team. R: A Language and Environment for Statistical Computing. 3.3.1 ed. Vienna, Austria: R Foundation for Statistical Computing; 2016.

[58] Linzer DA, Lewis JB. poLCA: An R package for polytomous variable latent class analysis. 2011. 2011;42(10):29. Epub 2011-06-14.

[59] Nylund KL, Asparouhov T, Muthén BO. Deciding on the number of classes in latent class analysis and growth mixture modeling: A Monte Carlo simulation study. Structural Equation Modeling: A Multidisciplinary Journal. 2007;14(4):535–69.

[60] Geurts K, Thomas I, Wets G. Understanding spatial concentrations of road accidents using frequent item sets. Accid Anal Prev. 2005;37(4):787–99. 10.1016/j.aap.2005.03.023 15899471

[61] Depaire B, Wets G, Vanhoof K. Traffic accident segmentation by means of latent class clustering. Accid Anal Prev. 2008;40(4):1257–66. 10.1016/j.aap.2008.01.007 18606254

[62] Kumar S, Toshniwal D. A data mining framework to analyze road accident data. Journal of Big Data. 2015;2(1):1–18.

[63] Shaheed MS, Gkritza K. A latent class analysis of single-vehicle motorcycle crash severity outcomes. Analytic Methods in Accident Research. 2014;2:30–8.

[64] Eluru N, Bagheri M, Miranda-Moreno LF, Fu L. A latent class modeling approach for identifying vehicle driver injury severity factors at highway-railway crossings. Accid Anal Prev. 2012;47:119–27. 10.1016/j.aap.2012.01.027 22342959

[65] de Oña J, López G, Mujalli R, Calvo FJ. Analysis of traffic accidents on rural highways using Latent Class Clustering and Bayesian Networks. Accid Anal Prev. 2013;51:1–10. 10.1016/j.aap.2012.10.016 23182777

[66] Habibovic A, Davidsson J. Requirements of a system to reduce car-to-vulnerable road user crashes in urban intersections. Accid Anal Prev. 2011;43(4):1570–80. 10.1016/j.aap.2011.03.019 21545892

[67] Konstantopoulos P, Chapman P, Crundall D. Driver's visual attention as a function of driving experience and visibility. Using a driving simulator to explore drivers' eye movements in day, night and rain driving. Accid Anal Prev. 2010;42(3):827–34. Epub 2010/04/13. 10.1016/j.aap.2009.09.022 20380909

[68] Martensen H, Focant N, Diependaele K. Let's talk about the weather–Interpretation of short term changes in road accident outcomes. Transportation Research Procedia. 2016;14:96–104.

[69] Miranda-Moreno L, Nosal T. Weather or not to cycle; whether or not cyclist ridership has grown: A look at weather's impact on cycling facilities and temporal trends in an urban environment. US Transportation Research Board Annual Conference; Washington, D.C. 2011.

[70] Meng M, Zhang J, Wong YD, Au PH. Effect of weather conditions and weather forecast on cycling travel behavior in Singapore. International Journal of Sustainable Transportation. 2016;10(9):773–80.

[71] Rose G, Ahmed F, Figliozzi M, Jakob C. Quantifying and comparing the effects of weather on bicycle demand in Melbourne (Australia) and Portland (USA). Transportation Research Board Annual Meeting, 90th; Washington, DC, USA2011.

[72] Van der Meel EM. Red light running by cyclists: Which factors influence the red light running by cyclists? Delft: Civil Engineering and Geosciences; 2013.

[73] Habibovic A, Davidsson J. Causation mechanisms in car-to-vulnerable road user crashes: Implications for active safety systems. Accid Anal Prev. 2012;49:493–500. 10.1016/j.aap.2012.03.022 23036427

[74] Vansteenkiste P, Van Hamme D, Veelaert P, Philippaerts R, Cardon G, Lenoir M. Cycling around a curve: The effect of cycling speed on steering and gaze behavior. PLoS ONE. 2014;9(7):e102792 10.1371/journal.pone.0102792 25068380 PMC4113223

[75] Koustanaï A, Boloix E, Van Elslande P, Bastien C. Statistical analysis of “looked-but-failed-to-see” accidents: Highlighting the involvement of two distinct mechanisms. Accid Anal Prev. 2008;40(2):461–9. 10.1016/j.aap.2007.08.001 18329395

[76] Räsänen M, Summala H. Attention and expectation problems in bicycle–car collisions: An in-depth study. Accid Anal Prev. 1998;30(5):657–66. 9678219 10.1016/s0001-4575(98)00007-4

[77] Summala H, Pasanen E, Räsänen M, Sievänen J. Bicycle accidents and drivers' visual search at left and right turns. Accid Anal Prev. 1996;28(2):147–53. 8703272 10.1016/0001-4575(95)00041-0

[78] Rissel C, Campbell F, Ashley B, Jackson L. Driver road rule knowledge and attitudes towards cyclists. Aust J Prim Health. 2002;8(2):66–9.

[79] Amsden M, Huber T. Bicycle crash analysis for Wisconsin using a crash typing tool (PBCAT) and geographic information system (GIS). Springfield VA: Wisconsin Department of Transportation and the Federal Highway Administration, 2006.

[80] van den Dool D, Murphy J. Cycling on rural roads. Journal of the Australasian College of Road Safety 2014;25(4):58–67.

[81] Wegman F, Zhang F, Dijkstra A. How to make more cycling good for road safety? Accid Anal Prev. 2012;44(1):19–29. 10.1016/j.aap.2010.11.010 22062332

[82] Twisk DAM, Stacey C. Trends in young driver risk and countermeasures in European countries. J Safety Res. 2007;38(2):245–57. Epub 2007/05/05. 10.1016/j.jsr.2007.03.006 17478195

[83] Twisk DAM, Vlakveld WP, Commandeur JJF, Shope JT, Kok G. Five road safety education programmes for young adolescent pedestrians and cyclists: A multi-programme evaluation in a field setting. Accid Anal Prev. 2014;66:55–61. 10.1016/j.aap.2014.01.002 24509322

[84] Li G, Braver ER, Chen LH. Fragility versus excessive crash involvement as determinants of high death rates per vehicle-mile of travel among older drivers. Accid Anal Prev. 2003;35(2):227–35. Epub 2002/12/31. 12504143 10.1016/s0001-4575(01)00107-5

[85] Anstey KJ, Wood J, Lord S, Walker JG. Cognitive, sensory and physical factors enabling driving safety in older adults. Clin Psychol Rev. 2005;25(1):45–65. Epub 2004/12/15. 10.1016/j.cpr.2004.07.008 15596080

[86] Hess G, Peterson MN. “Bicycles may use full lane” signage communicates U.S. roadway rules and increases perception of safety. PLoS ONE. 2015;10(8):e0136973 10.1371/journal.pone.0136973 26317355 PMC4552809

[87] Kwan I, Mapstone J. Interventions for increasing pedestrian and cyclist visibility for the prevention of death and injuries. Cochrane Database Syst Rev. 2006;(4):Cd003438 Epub 2006/10/21. 10.1002/14651858.CD003438.pub2 17054171 PMC8713592

[88] Fyhri A, Bjørnskau T, Backer-Grøndahl A. Bicycle helmets–A case of risk compensation? Transportation Research Part F: Traffic Psychology and Behaviour. 2012;15(5):612–24.

[89] Charlton SG. Conspicuity, memorability, comprehension, and priming in road hazard warning signs. Accid Anal Prev. 2006;38(3):496–506. 10.1016/j.aap.2005.11.007 16386230

[90] Nathanail E, Adamos G. Road safety communication campaigns: Research designs and behavioral modeling. Transportation Research Part F: Traffic Psychology and Behaviour. 2013;18:107–22.

[91] Yang Y, Sun J. Study on pedestrian red-time crossing behavior. Transportation Research Record: Journal of the Transportation Research Board. 2013;2393:117–24.

[92] Thomas B, DeRobertis M. The safety of urban cycle tracks: A review of the literature. Accid Anal Prev. 2013;52:219–27. 10.1016/j.aap.2012.12.017 23396201

[93] Pucher J, Buehler R. Making Cycling Irresistible: Lessons from The Netherlands, Denmark and Germany. Transport Reviews. 2008;28(4):495–528.

