# Supplementary material for: Characteristics of cyclist crashes in Italy using latent class analysis and association rule mining
Source: PLoS One. 2017 Feb 3;12(2):e0171484. doi: 10.1371/journal.pone.0171484 (PMC5291444; doi:10.1371/journal.pone.0171484)
Supplement: S1 Table — (DOCX) [file pone.0171484.s001.docx]

**S1 Table. Confusion Matrix**

| **Observed classes** | **C1** | **C2** | **C3** | **C4** | **C5** | **C6** | **C7** | **C8** | **C9** | **C10** | **C11** | **C12** | **C13** | **C14** | **C15** | **C16** | **C17** | **C18** | **C19** |
| --- | --- | --- | --- | --- | --- | --- | --- | --- | --- | --- | --- | --- | --- | --- | --- | --- | --- | --- | --- |
| **1** | 859 | 0 | 20 | 27 | 0 | 4 | 25 | 0 | 0 | 0 | 37 | 19 | 1 | 3 | 0 | 103 | 22 | 151 | 1 |
| **2** | 0 | 1165 | 0 | 4 | 0 | 43 | 0 | 16 | 0 | 0 | 1 | 0 | 0 | 9 | 41 | 0 | 0 | 0 | 8 |
| **3** | 52 | 0 | 5888 | 6 | 0 | 0 | 127 | 38 | 0 | 0 | 3 | 0 | 0 | 104 | 219 | 241 | 24 | 5 | 4 |
| **4** | 17 | 0 | 0 | 4773 | 0 | 26 | 2 | 12 | 0 | 0 | 313 | 0 | 5 | 11 | 17 | 5 | 1 | 82 | 0 |
| **5** | 0 | 0 | 0 | 0 | 413 | 3 | 0 | 0 | 4 | 8 | 0 | 0 | 0 | 0 | 0 | 0 | 0 | 0 | 1 |
| **6** | 7 | 27 | 0 | 18 | 3 | 1200 | 1 | 0 | 0 | 0 | 9 | 3 | 2 | 0 | 1 | 18 | 0 | 13 | 46 |
| **7** | 8 | 0 | 77 | 0 | 0 | 2 | 648 | 3 | 0 | 0 | 14 | 0 | 0 | 4 | 10 | 13 | 0 | 63 | 3 |
| **8** | 1 | 1 | 21 | 48 | 0 | 0 | 2 | 2231 | 0 | 0 | 33 | 0 | 0 | 97 | 26 | 3 | 0 | 0 | 0 |
| **9** | 0 | 0 | 0 | 0 | 9 | 0 | 0 | 0 | 1984 | 259 | 0 | 0 | 0 | 0 | 0 | 9 | 0 | 0 | 0 |
| **10** | 0 | 0 | 0 | 0 | 9 | 0 | 0 | 0 | 35 | 3406 | 0 | 0 | 0 | 0 | 0 | 0 | 0 | 0 | 0 |
| **11** | 16 | 1 | 2 | 181 | 0 | 6 | 11 | 29 | 0 | 0 | 2282 | 7 | 153 | 13 | 46 | 3 | 0 | 2 | 0 |
| **12** | 40 | 0 | 0 | 1 | 0 | 6 | 0 | 0 | 0 | 0 | 2 | 1590 | 86 | 0 | 0 | 32 | 11 | 3 | 0 |
| **13** | 5 | 0 | 0 | 8 | 0 | 3 | 1 | 0 | 0 | 0 | 36 | 8 | 519 | 0 | 0 | 0 | 0 | 2 | 0 |
| **14** | 3 | 1 | 43 | 30 | 0 | 0 | 1 | 212 | 0 | 0 | 18 | 0 | 0 | 1599 | 483 | 3 | 0 | 0 | 0 |
| **15** | 0 | 7 | 114 | 171 | 0 | 1 | 7 | 689 | 0 | 0 | 76 | 0 | 5 | 1032 | 5304 | 8 | 0 | 0 | 0 |
| **16** | 193 | 1 | 53 | 124 | 0 | 9 | 16 | 11 | 0 | 0 | 33 | 44 | 0 | 11 | 15 | 2758 | 0 | 413 | 1 |
| **17** | 47 | 0 | 5 | 0 | 0 | 3 | 4 | 0 | 0 | 0 | 0 | 1 | 0 | 0 | 0 | 0 | 662 | 42 | 0 |
| **18** | 257 | 0 | 0 | 110 | 0 | 14 | 99 | 0 | 0 | 0 | 8 | 3 | 0 | 0 | 0 | 291 | 29 | 3372 | 6 |
| **19** | 2 | 18 | 3 | 0 | 0 | 44 | 3 | 0 | 0 | 0 | 0 | 0 | 0 | 0 | 2 | 3 | 1 | 3 | 670 |
