# Supplementary material for: Characteristics of cyclist crashes in Italy using latent class analysis and association rule mining
Source: PLoS One. 2017 Feb 3;12(2):e0171484. doi: 10.1371/journal.pone.0171484 (PMC5291444; doi:10.1371/journal.pone.0171484)
Supplement: S2 Table — (DOCX) [file pone.0171484.s002.docx]

**Table S2. Latent Class Characteristics (Percentage of Cluster Observations)**

| **Variable** | **Category** | **C1** | **C2** | **C3** | **C4** | **C5** | **C6** | **C7** | **C8** | **C9** | **C10** | **C11** | **C12** | **C13** | **C14** | **C15** | **C16** | **C17** | **C18** | **C19** |  |
| --- | --- | --- | --- | --- | --- | --- | --- | --- | --- | --- | --- | --- | --- | --- | --- | --- | --- | --- | --- | --- | --- |
| **Percentage of the observations** | | 3.0 | 2.5 | 12.5 | 11.1 | 0.9 | 2.8 | 1.9 | 6.5 | 4.1 | 7.4 | 5.8 | 3.4 | 1.5 | 5.8 | 12.4 | 7.0 | 1.5 | 8.4 | 1.5 | |
| **Opponent vehicle** | |  |  |  |  |  |  |  |  |  |  |  |  |  |  |  |  |  |  |  | |
|  | **Car** | 65.0 | 90.2 | 82.2 | 81.3 | 0.0 | 78.9 | 59.8 | 90.3 | 0.0 | 0.0 | 69.9 | 84.3 | 77.9 | 84.5 | 86.2 | 79.7 | 81.6 | 76.8 | 89.3 |  |
|  | **Bus** | 0.4 | 0.0 | 1.4 | 0.9 | 0.0 | 0.9 | 1.1 | 0.2 | 0.0 | 0.0 | 0.6 | 0.4 | 1.3 | 0.0 | 0.4 | 1.1 | 0.4 | 2.3 | 0.7 |  |
|  | **Truck** | 12.2 | 6.2 | 6.3 | 8.5 | 0.0 | 7.2 | 5.9 | 6.5 | 0.0 | 0.0 | 8.6 | 9.6 | 9.3 | 6.2 | 6.7 | 5.1 | 9.1 | 5.2 | 5.7 |  |
|  | **PWT** | 7.5 | 1.0 | 8.2 | 6.2 | 0.0 | 5.9 | 31.5 | 1.1 | 0.0 | 0.0 | 9.9 | 0.7 | 1.0 | 3.0 | 4.1 | 8.5 | 3.1 | 13.9 | 3.0 |  |
|  | **Other vehicles** | 3.8 | 1.5 | 0.9 | 2.1 | 0.0 | 2.2 | 0.8 | 1.8 | 0.0 | 0.0 | 8.2 | 1.3 | 3.0 | 2.3 | 2.4 | 2.0 | 2.6 | 0.4 | 0.2 |  |
|  | **Multiple vehicles** | 11.0 | 1.1 | 1.2 | 1.0 | 0.0 | 4.0 | 0.8 | 0.1 | 0.0 | 0.0 | 2.9 | 3.8 | 7.5 | 4.0 | 0.2 | 3.4 | 3.2 | 1.4 | 0.9 |  |
|  | **No opponent vehicle** | 0.0 | 0.0 | 0.0 | 0.0 | 100.0 | 0.9 | 0.0 | 0.0 | 100.0 | 100.0 | 0.0 | 0.0 | 0.0 | 0.0 | 0.0 | 0.3 | 0.0 | 0.0 | 0.2 |  |
| **Cyclist’s gender** |  |  |  |  |  |  |  |  |  |  |  |  |  |  |  |  |  |  |  |  |  |
|  | **Male** | 91.0 | 66.4 | 69.3 | 67.2 | 70.6 | 67.5 | 65.5 | 66.3 | 70.4 | 70.7 | 79.4 | 58.1 | 57.9 | 91.3 | 54.0 | 62.9 | 69.2 | 68.1 | 68.3 |  |
|  | **Female** | 9.0 | 33.6 | 30.7 | 32.8 | 29.4 | 32.5 | 34.5 | 33.7 | 29.6 | 29.3 | 20.6 | 41.9 | 42.1 | 8.7 | 46.0 | 37.1 | 30.8 | 31.9 | 31.7 |  |
| **Cyclist’s age** |  |  |  |  |  |  |  |  |  |  |  |  |  |  |  |  |  |  |  |  |  |
|  | **0-14** | 3.1 | 1.5 | 13.9 | 4.38 | 2.6 | 4.7 | 11.7 | 1.6 | 6.1 | 4.7 | 3.4 | 3.5 | 1.6 | 2.6 | 3.6 | 6.7 | 3.7 | 15.8 | 7.1 |  |
|  | **15-24** | 7.9 | 14.7 | 16.9 | 10.5 | 9.1 | 13.6 | 13.0 | 11.4 | 13.7 | 9.6 | 10.0 | 10.2 | 10.7 | 7.6 | 10.7 | 11.8 | 9.1 | 15.2 | 15.0 |  |
|  | **25-44** | 30.9 | 39.2 | 26.5 | 29.2 | 31.9 | 40.2 | 18.6 | 32.4 | 26.2 | 27.5 | 28.8 | 31.2 | 35.4 | 33.4 | 28.0 | 28.1 | 35.6 | 26.5 | 33.7 |  |
|  | **45-54** | 21.1 | 18.3 | 11.9 | 16.5 | 20.4 | 16.2 | 11.6 | 18.5 | 17.7 | 18.9 | 19.3 | 17.1 | 18.2 | 21.2 | 15.1 | 15.1 | 17.3 | 10.4 | 14.6 |  |
|  | **55-64** | 15.7 | 10.8 | 8.5 | 13.6 | 14.6 | 9.3 | 11.7 | 16.8 | 14.3 | 14.1 | 13.2 | 13.7 | 14.3 | 15.3 | 13.6 | 12.3 | 15.6 | 8.2 | 8.1 |  |
|  | **65 and older** | 21.2 | 15.3 | 21.8 | 25.2 | 20.2 | 15.4 | 33.0 | 19.4 | 20.7 | 24.70 | 24.9 | 23.4 | 19.9 | 18.9 | 28.5 | 25.0 | 18.0 | 23.3 | 21.1 |  |
|  | **Not specified** | 0.1 | 0.2 | 0.5 | 0.7 | 1.1 | 0.5 | 0.4 | 0.1 | 1.4 | 0.6 | 0.5 | 0.9 | 0.0 | 1.1 | 0.4 | 1.1 | 0.8 | 0.6 | 0.4 |  |
| **Time of the day** |  |  |  |  |  |  |  |  |  |  |  |  |  |  |  |  |  |  |  |  |  |
|  | **Day time** | 81.4 | 76.8 | 80.1 | 85.9 | 75.7 | 74.8 | 85.9 | 84.6 | 84.2 | 79.7 | 75.6 | 85.0 | 86.0 | 79.0 | 86.4 | 82.5 | 82.4 | 80.8 | 76.0 |  |
|  | **Evening** | 15.8 | 20.6 | 18.0 | 13.1 | 17.4 | 21.8 | 12.8 | 14.4 | 13.9 | 15.8 | 19.3 | 14.0 | 13.2 | 18.0 | 12.7 | 15.0 | 15.6 | 17.7 | 21.7 |  |
|  | **Late night** | 1.9 | 2.3 | 1.8 | 0.7 | 6.3 | 2.85 | 0.5 | 0.9 | 1.9 | 3.8 | 4.8 | 0.6 | 0.8 | 2.3 | 0.8 | 1.8 | 1.3 | 1.5 | 2.4 |  |
|  | **Not specified** | 1.0 | 0.3 | 0.1 | 0.2 | 0.6 | 0.5 | 0.9 | 0.1 | 0.1 | 0.7 | 0.3 | 0.4 | 0.0 | 0.7 | 0..1 | 0.7 | 0.8 | 0.1 | 0.0 |  |
| **Cyclist’s maneuver** | |  |  |  |  |  |  |  |  |  |  |  |  |  |  |  |  |  |  |  |  |
|  | **Straight forward or normal driving** | 0.0 | 84.9 | 0.0 | 91.5 | 7.8 | 41.7 | 0.0 | 88.9 | 49.8 | 0.0 | 92.1 | 0.0 | 81.2 | 82.8 | 81.8 | 0.0 | 0.0 | 0.0 | 0.0 |  |
| **Not keeping a safe distance** | | 7.7 | 0.0 | 1.3 | 0.1 | 0.0 | 4.4 | 2.0 | 0.1 | 3.6 | 0.0 | 0.2 | 14.2 | 0.5 | 0.6 | 0.1 | 0.0 | 92.4 | 2.6 | 1.6 |  |
| **Ignoring stop signs or red traffic light** | | 0.0 | 0.6 | 22.3 | 0.0 | 0.0 | 0.0 | 0.0 | 0.2 | 1.2 | 0.0 | 0.0 | 0.0 | 0.0 | 0.9 | 0.3 | 0.0 | 0.0 | 0.0 | 20.6 |  |
| **Not respecting the right of way** | | 0.0 | 0.8 | 28.5 | 0.0 | 1.3 | 0.0 | 0.0 | 0.8 | 4.5 | 0.0 | 0.1 | 0.0 | 0.0 | 0.8 | 0.4 | 0.0 | 0.0 | 0.0 | 21.8 |  |
| **Driving in a forbidden direction or on opposite sides of road** | | 3.2 | 5.6 | 21.1 | 3.6 | 0.9 | 3.6 | 0.0 | 1.7 | 5.4 | 0.0 | 0.2 | 3.5 | 0.0 | 1.5 | 11.0 | 0.0 | 4.6 | 18.4 | 23.4 |  |
| **Traveling too fast** | | 7.8 | 0.2 | 1.5 | 0.2 | 4.6 | 1.3 | 0.0 | 0.3 | 6.2 | 2.9 | 0.1 | 9.0 | 1.1 | 1.0 | 0.1 | 0.0 | 3.1 | 2.9 | 2.7 |  |
|  | **Turning right** | 0.0 | 1.2 | 2.0 | 0.0 | 0.2 | 0.0 | 0.0 | 2.5 | 0.6 | 0.0 | 0.3 | 0.0 | 0.0 | 0.3 | 1.7 | 0.1 | 0.0 | 0.0 | 1.3 |  |
|  | **Turning left** | 0.4 | 2.8 | 4.3 | 0.0 | 0.0 | 2.0 | 77.0 | 3.2 | 1.6 | 0.0 | 1.6 | 0.0 | 0.0 | 2.9 | 2.6 | 0.7 | 0.0 | 1.7 | 5.7 |  |
|  | **Overtaking** | 3.3 | 0.1 | 1.2 | 1.0 | 0.0 | 0.1 | 1.0 | 0.0 | 0.9 | 0.0 | 0.3 | 0.1 | 0.1 | 1.4 | 0.0 | 0.0 | 0.0 | 1.0 | 1.4 |  |
| **Unknown or others** | | 77.6 | 4.0 | 17.9 | 3.6 | 85.2 | 46.9 | 20.0 | 2.3 | 26.2 | 97.1 | 5.3 | 73.3 | 17.2 | 8.0 | 2.0 | 99.2 | 0.0 | 73.4 | 21.5 |  |
| **Opponent vehicle maneuver** | |  |  |  |  |  |  |  |  |  |  |  |  |  |  |  |  |  |  |  |  |
|  | **Straight forward or normal driving** | 48.6 | 0.0 | 74.0 | 0.0 | 0.0 | 19.0 | 68.1 | 0.0 | 0.0 | 0.0 | 0.0 | 0.0 | 0.0 | 0.0 | 0.0 | 11.9 | 95.5 | 74.9 | 74.5 |  |
| **Not keeping a safe distance** | | 1.4 | 0.4 | 0.1 | 1.2 | 0.0 | 10.7 | 3.3 | 0.8 | 0.0 | 0.0 | 51.7 | 12.1 | 95.5 | 0.0 | 1.1 | 0.0 | 0.0 | 0.1 | 0.1 |  |
| **Ignoring stop signs or red traffic light** | | 0.0 | 23.1 | 0.6 | 0.0 | 0.0 | 0.0 | 0.0 | 3.8 | 0.0 | 0.0 | 0.0 | 0.0 | 0.0 | 27.6 | 26.2 | 0.0 | 0.1 | 0.0 | 0.9 |  |
| **Not respecting the right of way** | | 0.0 | 59.9 | 0.9 | 0.0 | 0.0 | 0.0 | 0.2 | 71.0 | 0.0 | 0.0 | 0.0 | 0.0 | 0.0 | 46.2 | 42.4 | 0.0 | 0.0 | 0.0 | 0.0 |  |
| **Driving in a forbidden direction or on opposite sides of road** | | 1.3 | 0.8 | 0.8 | 3.3 | 0.0 | 1.5 | 0.5 | 0.2 | 0.0 | 0.0 | 1.2 | 0.6 | 4.5 | 2.0 | 1.8 | 0.0 | 0.2 | 0.1 | 0.0 |  |
| **Traveling too fast** | | 4.9 | 0.7 | 2.2 | 6.1 | 0.0 | 6.8 | 10.0 | 1.3 | 0.0 | 0.0 | 14.7 | 7.1 | 0.0 | 0.6 | 1.5 | 1.4 | 0.0 | 3.2 | 1.7 |  |
|  | **Turning right** | 0.0 | 3.8 | 9.1 | 0.0 | 0.0 | 0.0 | 0.0 | 9.9 | 0.0 | 0.0 | 0.0 | 0.0 | 0.0 | 5.6 | 8.3 | 0.0 | 1.6 | 0.0 | 7.2 |  |
|  | **Turning left** | 1.8 | 4.4 | 9.7 | 10.3 | 0.0 | 3.4 | 0.4 | 0.2 | 0.0 | 0.0 | 0.7 | 0.0 | 0.0 | 13.9 | 7.8 | 1.6 | 0.6 | 0.0 | 12.7 |  |
| **Overtaking** | | 6.4 | 0.6 | 0.3 | 4.7 | 0.0 | 1.8 | 11.7 | 1.7 | 0.0 | 0.0 | 1.2 | 0.3 | 0.0 | 1.2 | 0.7 | 0.0 | 0.2 | 1.4 | 0.0 |  |
| **Unknown or others** | | 35.7 | 6.3 | 2.4 | 74.5 | 100 | 56.8 | 5.9 | 11.2 | 100 | 100 | 30.5 | 80.0 | 0.0 | 3.7 | 10.2 | 85.1 | 1.9 | 20.3 | 2.8 |  |
| **Day of the week** | |  |  |  |  |  |  |  |  |  |  |  |  |  |  |  |  |  |  |  |  |
|  | **Weekdays** | 66.1 | 81.4 | 80.2 | 80.6 | 67.7 | 78.5 | 80.5 | 80.8 | 74.0 | 71.4 | 72.3 | 81.5 | 83.5 | 67.5 | 85.5 | 82.0 | 79.0 | 82.4 | 77.5 |  |
|  | **Weekend** | 34.0 | 18.6 | 18.8 | 19.4 | 32.3 | 21.5 | 19.5 | 19.2 | 26.0 | 28.6 | 27.7 | 18.5 | 16.5 | 32.5 | 14.5 | 18.0 | 21.0 | 17.6 | 22.5 |  |
| **Season** |  |  |  |  |  |  |  |  |  |  |  |  |  |  |  |  |  |  |  |  |  |
|  | **Winter** | 16.9 | 32.0 | 14.5 | 14.8 | 23.8 | 30.2 | 13.6 | 15.6 | 12.6 | 14.8 | 14.2 | 15.5 | 15.7 | 18.2 | 17.2 | 15.4 | 13.5 | 12.0 | 28.8 |  |
|  | **Spring** | 31.1 | 18.5 | 31.7 | 31.5 | 25.0 | 17.6 | 31.6 | 27.9 | 34.6 | 32.5 | 28.3 | 31.2 | 28.8 | 29.6 | 27.8 | 28.6 | 34.0 | 34.0 | 21.7 |  |
|  | **Summer** | 36.6 | 8.3 | 34.2 | 32.3 | 14.2 | 9.3 | 36.7 | 30.7 | 37.1 | 35.3 | 36.6 | 30.4 | 30.2 | 37.0 | 29.8 | 35.2 | 28.1 | 33.9 | 8.9 |  |
|  | **Autumn** | 15.5 | 41.6 | 19.6 | 21.5 | 37.0 | 43.0 | 18.2 | 25.8 | 15.6 | 17.3 | 20.9 | 23.0 | 25.3 | 15.3 | 25.2 | 20.8 | 24.5 | 20.1 | 40.6 |  |
| **Road type** |  |  |  |  |  |  |  |  |  |  |  |  |  |  |  |  |  |  |  |  |  |
|  | **Urban municipal** | 31.6 | 85.8 | 85.3 | 78.4 | 88.7 | 79.7 | 69.1 | 74.4 | 85.7 | 79.5 | 58.9 | 89.7 | 94.3 | 56.9 | 92.8 | 83.8 | 80.5 | 85.6 | 86.3 |  |
| **Urban provincial, regional and national** | | 16.6 | 7.3 | 7.5 | 10.4 | 3.9 | 7.81 | 15.7 | 13.5 | 5.4 | 6.3 | 10.6 | 6.1 | 3.1 | 21.4 | 4.8 | 10.0 | 8.2 | 7.5 | 7.5 |  |
|  | **Rural** | 51.8 | 6.9 | 7.3 | 11.3 | 7.4 | 12.5 | 15.2 | 12.1 | 9.0 | 14.2 | 30.6 | 4.2 | 2.6 | 21.7 | 2.4 | 6.2 | 11.2 | 7.0 | 6.3 |  |
| **Pavement condition** | |  |  |  |  |  |  |  |  |  |  |  |  |  |  |  |  |  |  |  |  |
|  | **Dry** | 96.1 | 0.0 | 99.8 | 97.9 | 0.0 | 0.0 | 97.7 | 99.5 | 97.3 | 96.4 | 98.0 | 98.1 | 96.0 | 99.5 | 99.0 | 98.1 | 96.8 | 97.9 | 0.0 |  |
|  | **Wet** | 2.1 | 97.2 | 0.0 | 1.8 | 97.2 | 98.2 | 1.9 | 0.4 | 1.6 | 0.2 | 1.4 | 1.7 | 3.6 | 0.3 | 0.8 | 1.3 | 2.8 | 2.0 | 97.9 |  |
| **Slippery, frozen or snowy** | | 1.8 | 2.8 | 0.2 | 0.3 | 2.8 | 1.8 | 0.4 | 0.1 | 1.1 | 3.4 | 0.6 | 0.2 | 0.4 | 0.2 | 0.2 | 0.6 | 0.5 | 0.1 | 2.1 |  |
| **Weather** | |  |  |  |  |  |  |  |  |  |  |  |  |  |  |  |  |  |  |  |  |
|  | **Clear** | 94.6 | 20.6 | 94.8 | 95.1 | 31.4 | 10.4 | 95.0 | 93.2 | 95.9 | 95.2 | 94.8 | 95.5 | 96.3 | 94.4 | 93.5 | 92.9 | 94.9 | 95.1 | 25.6 |  |
|  | **Foggy** | 0.3 | 3.4 | 0.3 | 0.2 | 1.9 | 4.9 | 0.2 | 0.5 | 0.2 | 0.3 | 0.2 | 0.4 | 0.3 | 0.1 | 0.4 | 0.4 | 0.0  44 | 0.3 | 3.1 |  |
|  | **Rainy** | 0.0 | 62.0 | 0.0 | 0.0 | 53.7 | 70.2 | 0.0 | 0.0 | 0.0 | 0.0 | 0.0 | 0.0 | 0.0 | 0.0 | 0.0 | 0.0 | 0.0 | 0.0 | 58.7 |  |
| **Hail, Snow, Strong wind, other** | | 5.1 | 14.0 | 5.0 | 4.8 | 13.0 | 14.6 | 4.8 | 6.3 | 3.8 | 4.5 | 5.0 | 4.1 | 3.4 | 5.5 | 6.1 | 6.7 | 5.1 | 4.7 | 12.6 |  |
| **Road Signage** | |  |  |  |  |  |  |  |  |  |  |  |  |  |  |  |  |  |  |  |  |
|  | **Absent** | 12.1 | 4.5 | 8.4 | 9.2 | 13.0 | 6.1 | 8.1 | 0.6 | 14.0 | 12.8 | 9.9 | 9.2 | 5.7 | 9.8 | 7.0 | 7.2 | 9.4 | 8.6 | 6.0 |  |
|  | **Vertical** | 12.5 | 4.5 | 7.6 | 5.9 | 7.8 | 6.1 | 5.0 | 3.3 | 9.5 | 7.5 | 8.0 | 46.9 | 4.7 | 8.0 | 5.0 | 7.8 | 5.5 | 5.8 | 5.6 |  |
|  | **Horizontal** | 14.4 | 2.5 | 3.4 | 13.3 | 8.0 | 11.4 | 14.2 | 2.3 | 9.5 | 9.1 | 12.5 | 12.0 | 14.4 | 4.6 | 2.4 | 6.0 | 10.2 | 14.8 | 3.2 |  |
| **Vertical and horizontal** | | 61.0 | 88.5 | 80.7 | 71.6 | 71.2 | 76.4 | 72.7 | 93.8 | 66.9 | 70.6 | 69.6 | 74.1 | 75.2 | 77.6 | 85.6 | 79.0 | 75.0 | 70.8 | 85.2 |  |
| **Type of collision** | |  |  |  |  |  |  |  |  |  |  |  |  |  |  |  |  |  |  |  |  |
|  | **Head-on collision** | 11.5 | 4.7 | 7.8 | 7.6 | 2.6 | 6.7 | 1.4 | 2.0 | 9.9 | 1.3 | 4.0 | 0.0 | 0.5 | 7.7 | 8.1 | 6.8 | 1.1 | 10.8 | 9.6 |  |
|  | **Side-impact** | 55.1 | 93.8 | 90.8 | 90.4 | 3.0 | 68.5 | 89.8 | 95.5 | 27.1 | 2.2 | 31.3 | 0.4 | 11.2 | 90.5 | 90.9 | 84.7 | 17.8 | 87.0 | 89.4 |  |
|  | **Rear-end collision** | 28.8 | 1.6 | 1.4 | 2.0 | 0.4 | 15.7 | 8.9 | 2.5 | 4.7 | 0.5 | 64.5 | 5.2 | 13.5 | 1.8 | 1.0 | 7.6 | 35.9 | 2.1 | 1.1 |  |
|  | **Hit pedestrian** | 0.0 | 0.0 | 0.0 | 0.0 | 1.6 | 0.0 | 0.0 | 0.0 | 12.4 | 0.0 | 0.0 | 0.0 | 0.0 | 0.0 | 0.0 | 0.0 | 0.0 | 0.0 | 0.0 |  |
|  | **Hit stopped vehicle** | 4.5 | 0.0 | 0.0 | 0.0 | 0.0 | 9.1 | 0.0 | 0.0 | 1.3 | 0.1 | 0.3 | 94.5 | 69.5 | 0.0 | 0.0 | 1.0 | 45.2 | 0.0 | 0.0 |  |
|  | **Hit parked vehicle or object** | 0.0 | 0.0 | 0.0 | 0.0 | 13.8 | 0.0 | 0.0 | 0.0 | 44.7 | 4.3 | 0.0 | 0.0 | 0.0 | 0.0 | 0.0 | 0.0 | 0.0 | 0.0 | 0.0 |  |
|  | **Run-off-the-road** | 0.0 | 0.0 | 0.0 | 0.0 | 44.7 | 0.0 | 0.0 | 0.0 | 0.0 | 46.8 | 0.0 | 0.0 | 0.0 | 0.0 | 0.0 | 0.0 | 0.0 | 0.0 | 0.0 |  |
|  | **Other (no vehicle was involved)** | 0.0 | 0.0 | 0.0 | 0.0 | 33.9 | 0.0 | 0.0 | 0.0 | 0.0 | 44.9 | 0.0 | 0.0 | 0.0 | 0.0 | 0.0 | 0.0 | 0.0 | 0.0 | 0.0 |  |
| **Road segment** |  |  |  |  |  |  |  |  |  |  |  |  |  |  |  |  |  |  |  |  |  |
|  | **Crossroads** | 13.7 | 73.3 | 91.3 | 4.7 | 24.4 | 20.8 | 44.9 | 29.6 | 25.7 | 19.0 | 23.3 | 10.8 | 10.6 | 95.8 | 99.9 | 48.3 | 19.9 | 0.0 | 78.2 |  |
|  | **Not at junction** | 82.5 | 0.0 | 0.0 | 94.0 | 70.5 | 73.4 | 52.5 | 0.0 | 71.9 | 78.0 | 69.7 | 88.8 | 89.4 | 0.0 | 0.0 | 39.8 | 77.5 | 99.8 | 10.5 |  |
|  | **Roundabouts** | 3.8 | 26.7 | 8.8 | 1.3 | 5.1 | 5.83 | 2.6 | 70.4 | 2.4 | 3.0 | 7.1 | 0.4 | 0.0 | 4.2 | 0.1 | 11.9 | 2.6 | 0.2 | 11.3 |  |
|  |  |  |  |  |  |  |  |  |  |  |  |  |  |  |  |  |  |  |  |  |  |

Total percentage may not sum to 100 due to rounding
