# Supplementary material for: Characteristics of cyclist crashes in Italy using latent class analysis and association rule mining
Source: PLoS One. 2017 Feb 3;12(2):e0171484. doi: 10.1371/journal.pone.0171484 (PMC5291444; doi:10.1371/journal.pone.0171484)
Supplement: S3 Table — (DOCX) [file pone.0171484.s003.docx]

**S3 Table. Rules with Bicycle Fatalities as Consequent**

| **Antecedent** | **Support %** | **Confidence %** | **Lift** |
| --- | --- | --- | --- |
| **C1** |  |  |  |
| **Time of the day=Evening and Type of collision=Rear-end** | 6.52 | 20.48 | 3.14 |
| **Time of the day=Evening and Opponent vehicle=Car** | 10.92 | 15.83 | 2.43 |
| **Cyclist's age=>=65 and Day of the week= Weekdays** | 13.51 | 15.70 | 2.41 |
| **Time of the day=Evening and Season=Spring** | 5.18 | 13.64 | 2.09 |
| **Time of the day=Evening and Road signage =Vertical and horizontal** | 9.27 | 13.56 | 2.07 |
| **C2** |  |  |  |
| **Cyclist's age=>=65 and Season=Autumn** | 7.15 | 5.43 | 8.74 |
| **Cyclist's age=>=65 and Cyclist's gender=Male** | 9.72 | 4.00 | 6.43 |
| **Cyclist's age=>=65 and Cyclist’s maneuver=Straight forward** | 13.30 | 3.51 | 5.64 |
| **Cyclist's age=>=65 and Road signage=Vertical and horizontal** | 13.76 | 3.39 | 5.44 |
| **Cyclist's age=>=65 and Opponent vehicle maneuver=Not respecting the right of way** | 9.25 | 3.36 | 5.40 |
| **C3** |  |  |  |
| **Road type=Rural and Opponent vehicle=Car** | 5.50 | 9.21 | 6.06 |
| **Road type=Rural and Cyclist's gender=Male** | 5.51 | 9.19 | 6.05 |
| **Road type=Rural and Location type=Crossroad** | 6.10 | 9.05 | 5.95 |
| **Road type=Rural and Weather=Clear** | 6.45 | 8.55 | 5.62 |
| **Road type=Rural** | 6.89 | 8.42 | 5.54 |
| **C4** |  |  |  |
| **Type of collision=Head-on and Cyclist's gender=Male** | 5.64 | 4.71 | 3.70 |
| **Road type=Rural and Road signage=Vertical and horizontal** | 8.42 | 4.51 | 3.55 |
| **Season=Autumn and Cyclist's age=>=65** | 5.17 | 4.41 | 3.47 |
| **Road type=Rural and Opponent vehicle maneuver=Unknown or others** | 8.27 | 3.91 | 3.07 |
| **Opponent vehicle=Truck and Cyclist's gender=Male** | 5.87 | 3.88 | 3.05 |
| **C5** |  |  |  |
| **Road type=Rural and Cyclist's gender= Male** | 6.07 | 11.54 | 12.35 |
| **Road type=Rural and Location type=Not at junction** | 6.54 | 10.71 | 11.46 |
| **Road type=Rural and Cyclist’s maneuver =Unknown or others** | 6.78 | 10.34 | 11.07 |
| **Road type=Rural** | 7.48 | 9.38 | 10.03 |
| **Cyclist's age=45-54 and Weather=Clear** | 6.54 | 7.14 | 7.64 |
| **C6** |  |  |  |
| **Opponent vehicle maneuver=Traveling too fast and Time of the day=Daytime** | 5.03 | 10.29 | 6.05 |
| **Road type=Rural and Cyclist’s maneuver =Unknown or others** | 5.11 | 10.14 | 5.96 |
| **Road type=Rural and Cyclist's age=25-44** | 6.00 | 9.88 | 5.80 |
| **Road type =Rural and Day of the week=Weekdays** | 9.40 | 9.45 | 5.55 |
| **Road type=Rural and Opponent vehicle=Car** | 9.40 | 9.45 | 5.55 |
| **C7** |  |  |  |
| **Day of the week=Weekend and Cyclist's age=>=65** | 6.75 | 10.53 | 5.23 |
| **Day of the week=Weekend and Opponent vehicle=Car** | 11.14 | 7.45 | 3.70 |
| **Day of the week=Weekend and Time of the day=Daytime** | 16.23 | 6.57 | 3.26 |
| **Road type=Rural and Cyclist's gender=Female** | 5.57 | 6.38 | 3.17 |
| **Day of the week=Weekend and Location type=Crossroads** | 9.36 | 6.33 | 3.14 |
| **C8** |  |  |  |
| **Opponent vehicle=Truck and Cyclist’s maneuver =Straight forward or normal driving** | 5.81 | 2.80 | 5.74 |
| **Opponent vehicle=Truck and Day of the week=Weekdays** | 5.93 | 2.74 | 5.62 |
| **Opponent vehicle=Truck and Time of the day=Daytime** | 6.18 | 2.63 | 5.40 |
| **Opponent vehicle=Truck and Weather=Clear** | 6.18 | 2.63 | 5.40 |
| **Opponent vehicle=Truck and Type of collision=Side-impact** | 6.18 | 2.63 | 5.40 |
| **C9** |  |  |  |
| **Road type=Urban** | 5.40 | 3.28 | 4.36 |
| **Road signage=Absent and Cyclist's gender=Male** | 10.26 | 3.02 | 4.01 |
| **Road signage=Absent and Type of collision=Hit parked vehicle or object** | 5.88 | 3.01 | 4.00 |
| **Cyclist's age=>=65 and Cyclist's gender=Male** | 13.93 | 2.86 | 3.80 |
| **Road type=Rural and Location type=Not at junction** | 6.41 | 2.76 | 3.67 |
| **C10** |  |  |  |
| **Road type=Rural and Day of the week=Weekdays** | 9.13 | 7.94 | 3.26 |
| **Cyclist's age=>=65 and Cyclist's gender=Male** | 17.74 | 7.68 | 3.15 |
| **Road type=Rural and Cyclist's gender=Male** | 12.72 | 7.06 | 2.90 |
| **Cyclist's age=>=65 and Day of the week=Weekend** | 6.06 | 6.70 | 2.75 |
| **Road type=Rural and Road signage=Vertical and horizontal** | 8.41 | 6.55 | 2.69 |
| **C11** |  |  |  |
| **Opponent vehicle maneuver=Traveling too fast and Road type=Rural** | 5.20 | 20.98 | 3.72 |
| **Cyclist's age=>=65 and Road type=Rural** | 6.25 | 19.19 | 3.41 |
| **Location type=Crossroads and Road type=Rural** | 6.69 | 17.39 | 3.09 |
| **Time of the day=Evening and Road type=Rural** | 6.54 | 16.67 | 2.96 |
| **Opponent vehicle maneuver=Traveling too fast and Opponent vehicle=Car** | 8.32 | 16.16 | 2.87 |
| **C12** |  |  |  |
| **Road type=Urban provincial, regional and national and Cyclist’s maneuver=Unknown or others** | 5.08 | 3.33 | 5.37 |
| **Road type=Urban provincial, regional and national and Time of the day=Daytime** | 5.36 | 3.16 | 5.09 |
| **Road type=Urban provincial, regional and national and Pavement condition=Dry** | 6.09 | 2.78 | 4.47 |
| **Road type=Urban provincial, regional and national and Type of collision=Hit stopped vehicle** | 6.21 | 2.72 | 4.39 |
| **Location type=Crossroads and Cyclist's gender=Male** | 6.94 | 2.44 | 3.93 |
| **C13** |  |  |  |
| **Cyclist's age=>=65 and Season=Spring** | 5.51 | 3.13 | 9.08 |
| **Type of collision=Head-on and Day of the week=Weekdays** | 5.68 | 3.03 | 8.80 |
| **Type of collision=Head-on and Location type=Crossroads** | 6.37 | 2.70 | 7.85 |
| **Type of collision=Head-on and Weather=Clear** | 6.37 | 2.70 | 7.85 |
| **Type of collision=Head-on and Opponent vehicle maneuver=Not keeping a safe distance** | 6.54 | 2.63 | 7.64 |
| **C14** |  |  |  |
| **Opponent vehicle=Truck and Time of the day=Daytime** | 5.35 | 4.69 | 4.01 |
| **Type of collision=Head-on and Cyclist’s maneuver =Straight forward or normal driving** | 6.35 | 4.61 | 3.94 |
| **Opponent vehicle=Truck and Type of collision=Side-impact** | 5.68 | 4.41 | 3.77 |
| **Opponent vehicle=Truck and Weather=Clear** | 5.93 | 4.23 | 3.61 |
| **Opponent vehicle=Truck and Cyclist's gender=Male** | 6.02 | 4.17 | 3.56 |
| **C15** |  |  |  |
| **Opponent vehicle=Truck and Cyclist’s maneuver =Straight forward or normal driving** | 5.73 | 4.71 | 5.91 |
| **Opponent vehicle=Truck and Road signage=Vertical and horizontal** | 5.61 | 4.57 | 5.74 |
| **Opponent vehicle=Truck and Weather=Clear** | 6.11 | 4.42 | 5.55 |
| **Opponent vehicle=Truck and Time of the day=Weekdays** | 6.12 | 4.41 | 5.53 |
| **Opponent vehicle=Truck and Pavement condition=Dry** | 6.56 | 4.32 | 5.43 |
| **C16** |  |  |  |
| **Opponent vehicle=Truck** | 5.11 | 5.85 | 3.78 |
| **Road type=Rural** | 5.68 | 5.26 | 3.40 |
| **Cyclist's age=>=65 and Cyclist's gender=Male** | 16.89 | 5.14 | 3.32 |
| **Cyclist's age=>=65 and Location type=Not at junction** | 12.74 | 4.48 | 2.89 |
| **Cyclist's age=>=65 and Day of the week=Weekdays** | 21.94 | 4.46 | 2.88 |
| **C17** |  |  |  |
| **Cyclist's age=>=65 and Season=Autumn** | 5.36 | 7.32 | 4.66 |
| **Day of the week=Weekend and Season=Autumn** | 5.49 | 7.14 | 4.55 |
| **Cyclist's age=>=65 and Type of collision=Rear-end** | 5.75 | 6.82 | 4.35 |
| **Road type=Rural and Type of collision=Rear-end** | 6.54 | 6.00 | 3.83 |
| **Road signage=Absent and Location type=Not at junction** | 7.06 | 5.56 | 3.54 |
| **C18** |  |  |  |
| **Road type=Rural** | 5.99 | 6.37 | 3.61 |
| **Cyclist's age=>=65 and Cyclist's gender=Male** | 16.36 | 5.69 | 3.22 |
| **Cyclist's age=>=65 and Cyclist’s maneuver =Unknown or others** | 17.65 | 5.41 | 3.06 |
| **Cyclist's age=>=65 and Season=Spring** | 7.69 | 5.28 | 2.99 |
| **Cyclist's age=>=65 and Road signage=Vertical and horizontal** | 16.38 | 5.25 | 2.97 |
| **C19** |  |  |  |
| **Road type=Rural** | 6.15 | 6.52 | 6.10 |
| **Day of the week=Weekend and Weather=Clear** | 5.48 | 4.88 | 4.56 |
| **Cyclist's age=>=65 and Cyclist’s maneuver=Not respecting the right of way** | 5.75 | 4.65 | 4.35 |
| **Cyclist’s maneuver=Not respecting the right of way and Weather=Clear** | 6.55 | 4.08 | 3.82 |
| **Cyclist's age=>=65 and Cyclist's gender=Male** | 13.64 | 3.92 | 3.67 |
